# Single-, Dual-, and Multi-Stimuli-Responsive Nanogels for Biomedical Applications

**DOI:** 10.3390/gels10010061

**Published:** 2024-01-14

**Authors:** Naveen Kumar, Sauraj Singh, Piyush Sharma, Bijender Kumar, Anuj Kumar

**Affiliations:** 1 Department of Chemistry, S.D. College Muzaffarnagar, Muzaffarnagar 251001, Uttar Pradesh, India; 2 College of Pharmacy, Gachon University, Incheon 13120, Republic of Korea; saurajpolymeriitd@gmail.com; 3 Department of Zoology, S.D. College Muzaffarnagar, Muzaffarnagar 251001, Uttar Pradesh, India; piyushsharmasdcollege@gmail.com; 4 Creative Research Center for Nanocellulose Future Composites, Department of Mechanical Engineering, Inha University, Incheon 22212, Republic of Korea; bijenderkumarchem@gmail.com; 5 School of Materials Science and Technology, Indian Institute of Technology (BHU), Varanasi 221005, Uttar Pradesh, India

**Keywords:** nanogels, stimuli-responsiveness, nanomedicine, tissue engineering, drug delivery

## Abstract

In recent years, stimuli-responsive nanogels that can undergo suitable transitions under endogenous (e.g., pH, enzymes and reduction) or exogenous stimuli (e.g., temperature, light, and magnetic fields) for on-demand drug delivery, have received significant interest in biomedical fields, including drug delivery, tissue engineering, wound healing, and gene therapy due to their unique environment-sensitive properties. Furthermore, these nanogels have become very popular due to some of their special properties such as good hydrophilicity, high drug loading efficiency, flexibility, and excellent biocompatibility and biodegradability. In this article, the authors discuss current developments in the synthesis, properties, and biomedical applications of stimulus-responsive nanogels. In addition, the opportunities and challenges of nanogels for biomedical applications are also briefly predicted.

## 1. Introduction

Nanogels, also known as hydrogel nanoparticles (HNPs), are composed of cross-linked hydrophilic polymers and water and have an average size of about 100 nm. They have a highwater content, a large specific surface area, and good stability [1]. Nanogel-based systems are specifically designed to achieve the cargo’s long circulation half-life in the body as well as the ability to transport that cargo to the desired location in biomedical applications [2]. Furthermore, sustained and controlled on-demand drug release is another important issue in various therapeutic applications. Therefore, environmentally responsive nanogels have recently attracted significant attention. These nanogels, known as stimulus-sensitive or environmentally sensitive nanogels, can respond to an external stimulus by changing their physicochemical properties such as volume, water content, refractive index, internal network permeability, and hydrophobicity [3,4]. These stimuli can be classified into physical stimuli, which include changes in temperature, light and magnetic fields, and chemical stimuli, which include changes in pH, ionic strength as well as chemical or biological agents.

To achieve better therapeutic effects and also reduce unwanted side effects, compared with conventional nanogels, stimulus-responsive nanogels that release drugs or biologically active ingredients at the target site have been widely used in various biomedical applications. This is due to the fact that stimuli-responsive nanogels not only have drug delivery functions similar to other polymer nanoparticles, such as the ability to overcome biological barriers, protect drugs from quick degradation in biological systems, and provide a large surface area to conjugate target ligands, but they also have unique properties [5,6]. Specifically, (i) stimuli-responsive nanogels with a hydrophilic internal network can load and protect small hydrophilic molecules or biological macromolecular drugs; (ii) stimuli-responsive nanogels have a higher stability for prolonged circulation in the blood due to their chemically cross-linked structure; (iii) external stimuli can be used to adjust the drug loading and release profiles of stimulus-responsive nanogels, which can significantly improve loading efficiency and bioavailability and reduce any abnormalities or unwanted side effects; (iv) some stimulus-responsive nanogels, such as magnetic field-responsive nanogels, can use external stimuli to actively target specific locations; (v) stimulus-responsive nanogels have a better chance of specific retention at the target disease site because they are soft nanocarriers with the ability to flatten on the vascular surface and simultaneously anchor at multiple points. Several excellent reviews on the use of stimuli-responsive nanogels have recently been published [6,7,8].While some of the articles cover the variety of different stimuli used to release a therapeutic moiety, some of them provide a more in-depth discussion about the design and synthetic strategies of just one specific type of stimulation such as temperature-sensitive, light-sensitive, pH-sensitive, or redox-sensitive nanogels [9,10].The authors will concentrate on stimuli-responsive nanogels for drug delivery and provide an up-to-date overview of their preparation and potential pharmaceutical applications in this paper.

## 2. Classifications of Nanogels

**Figure 1:**
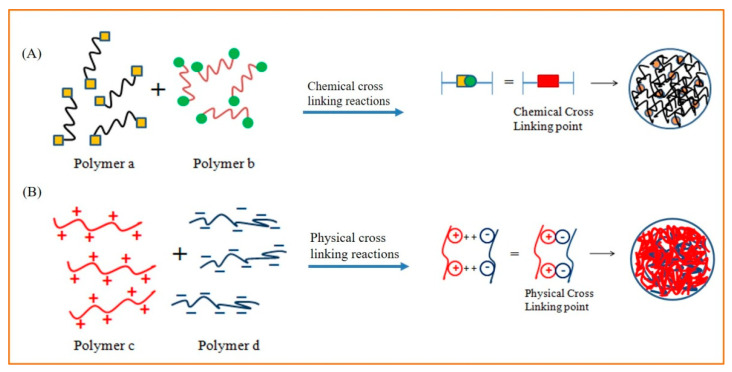
Schematic representation of (**A**) chemical and (**B**) physical cross-linking reactions during nanogel preparation.

On the basis of their cross-linked structure, nanogels are classified into two categories: one is the physically cross-linked nanogels and the other is the chemically cross-linked nanogels. Physically cross-linked nanogels self-assemble through weaker linkages such as non-covalent bonds (van der Waals, electrostatic, hydrogen bonds, and hydrophobic interactions), while chemically cross-linked nanogels form cross-links through covalent bonds, as shown in Figure 1.

**Figure 2:**
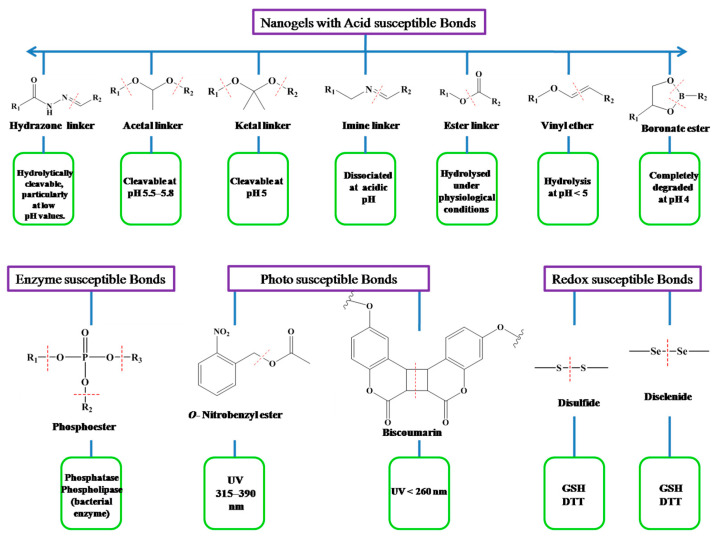
Linkers and their cleavage susceptibility conditions.

**Figure 3:**
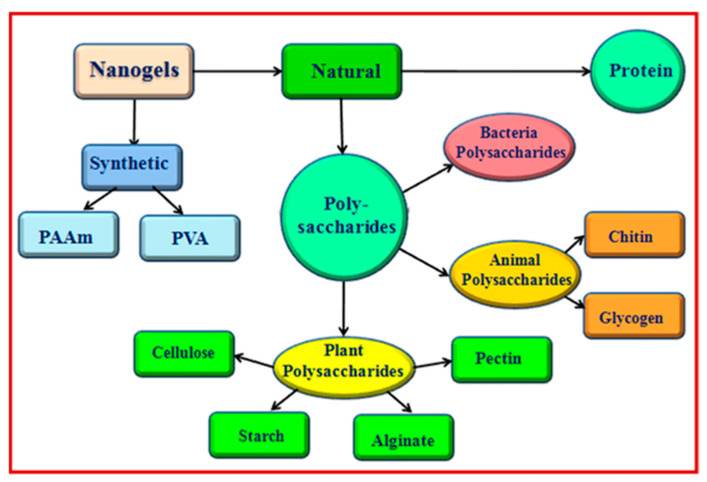
Classification of materials used in nanogel synthesis.

Chemically cross-linked nanogels have better physical and chemical stability than physically cross-linked nanogels because they are formed by covalent bonds, whereas the stability of physically cross-linked nanogels is sensitive and could lead to sol–gel transitions as a result of environmental stimuli changes. The covalent cross-linking strategy is extremely advantageous for controlling nanogel morphology, strength, and swellability, which are always necessary for the controlled loading and programmed delivery of therapeutic and theranostic agents [11,12]. When the nanogel reaches the target site, it must be cleaved in order for the active drugs to be released and the therapeutic effect to be achieved. Labile linkers are thus incorporated into nanogel scaffolds. The covalently bonded nanogels degrade when exposed to a specific stimulus. The structures and sensitivity conditions for various important linkers are listed in Figure 2 [3,13]. According to Zhao et al. [14], hydrogels can be divided into two types based on their source: synthetic polymer-based hydrogels and natural polymer-based hydrogels (Figure 3). The following sections below discuss some significant nanogel systems derived from both natural and artificial sources.

## 3. Synthesis Aspects of Stimuli-Responsive Nanogels

**Figure 4:**
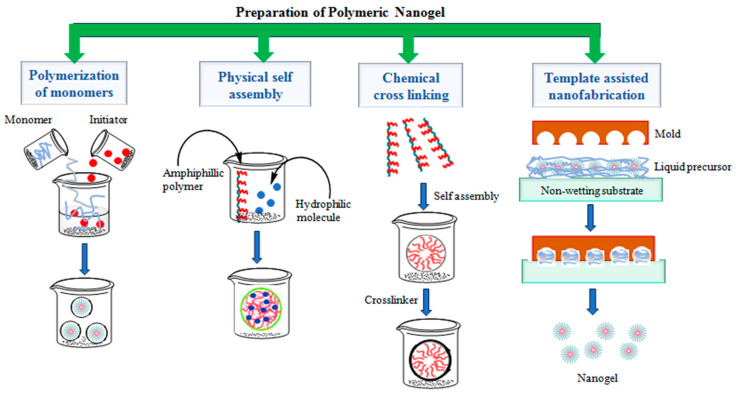
Schematic representation of different nanogel preparation methods.

There are three main types of nanogel synthesis methods: (1) the polymerization of monomers, (2) the physical or chemical cross-linking of polymer precursors or natural polymers, and (3) template-assisted nanofabrication [15]. A schematic representation of different nanogel preparation methods is presented in Figure 4.

**Figure 5:**
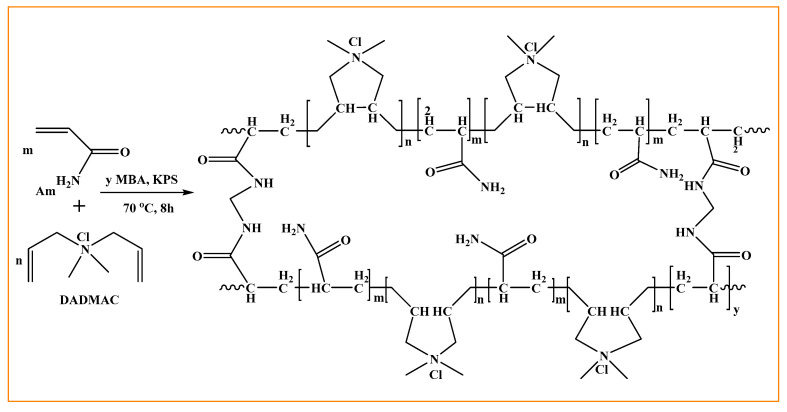
Schematic representation of cross-linked copolymer network structure of poly (Am-co-DADMAC) with NN-MBA.

The nanoscale of nanogels can be shaped by two main methods: “from top to bottom” and “from bottom to top”. In the “top-down” method, nanogels are created from large particles or clusters using physical, chemical, or mechanical methods [16]. For example, Rolland and colleagues developed a top-down method called Particle Replication in Non-Wetting Templates (PRINT) for creating polymer particles. In this method, the liquid precursor is kept inside non-wetted molds. This photolithography technique uses nonwetting elastomer molds of low-surface-energy perfluoropolyether networks prepared on patterned silicon templates by the photochemical cross-linking of dimethacrylate-functionalized perfluoropolyether oligomers. The use of molds allows for tight control over the size, shape, composition, and function of the particles and also eliminates the formation of interfacial films between the molded objects, thus creating mono-dispersed particles with a good uniformity in size and shape and allows for the transport of delicate cargo including pharmaceuticals and biological macromolecules [17]. Gallo et al. recently created peptide-based HGs and NGs using the hydrogelator Fmoc-FF alone or in two different ratios with (FY)3 peptide or its PEGylated analogue PEG8-(FY)3. Starting with the corresponding HGs, NGs were created using a top-down approach in which the macroscopic hydrogel was submicronized and stabilized with commercially available biocompatible surfactants. Both NGs and HGs can efficiently encapsulate Dox due to the common structure of their inner peptidic network. Cytotoxicity assays on the MDA-MB-231 breast cancer cell line revealed that the empty HGs and NGs had a high cell viability (>95%) and the Dox-loaded HGs and NGs had a lower cell viability (49–57%). The hydrogel peptide composition clearly influences the gelation kinetics (from 24 to 40 min) and drug release (16–28% after 72 h) from hydrogels. Similarly, in terms of net charges, the DLC values (0.137 and 0.093 for pure and mixed NGs, respectively) and release percentages (20–40% after 72 h) in NGs are affected by their composition [18]. Rosa et al., in one example, prepared peptide nanogel formulations based on the well-known hydrogelator Fmoc-FF using three different methods: water/oil emulsion (W/O), top-down, and water nanogelling. The top-down methodology has several advantages in this case. First, it avoids the use of mineral oil during preparation. As a result, organic solvents such as n-hexane can be avoided when extracting the nanogel solution. Furthermore, the top-down method necessitates only a few steps in preparation. Thus, the simple procedure, together with the high biocompatibility, are useful characteristics in the context of optimizing and simplifying their industrial fabrication. Furthermore, the nanoparticles produced by this method have a diameter of about 200 nm, making them suitable for any clinical application [19]. The “bottom-up” approach is achieved by designing molecular structures and assemblies from molecules or clusters that are then cross-linked by chemical or physical bonds [16]. Shimoda et al., for example, created nanogels by reacting a thiol-modified poly(ethylene glycol) (PEGSH) with an acryloyl-modified cholesterol-bearing pullulan (CHPOA). By varying the nanogel concentration, degree of substitution of the acryloyl groups in the CHPOA nanogels, and acryloyl:thiol molar ratio, the size of the nanogel assemblies was controlled in the range of 50–150 nm. They reported that the synthesized hybrid CHPOA–PEGSH nanogels are expected to be used as injectable nanocarriers capable of the long-term controlled release of proteins such as cytokines [20]. The authors Sekine et al. synthesized a biodegradable hydrogel by cross-linking a four-armed PEGSH and an acryloyl-group-modified nanogel (CHPOA) to create nanogels and nanogel-coated liposomes as building blocks. The nanogels can encapsulate a variety of hydrophobic substances, including drugs, proteins, and DNA, and exhibit molecular chaperone-like activity [21]. Monomer polymerization and preformed polymer chemical cross-linking in heterogeneous colloidal media, especially in water-in-oil inverse microemulsions, are commonly used methods for the preparation of stimuli-responsive nanogels. This method uses the charge imparted by the initiator to stabilize the nanogels, and nanogel synthesis occurs through the nucleation of water-soluble monomers, leading to the formation of a colloidal suspension. Small molecule drugs and biological macromolecules are easily trapped in nanogels using this technique. However, complex purification procedures and potential contamination by surfactants and organic solvents may restrict the use of formed nanogels for drug delivery. Under mild conditions and in aqueous media, the physical self-assembly of polymers has been used to produce various types of nanogels; thus, this method allows for the encapsulation of biologically active macromolecules [5]. Peptide and polysaccharide self-assembled nanogels make great options for bioactive delivery vehicles. In this case, Tai et al. created extracellular matrix-mimicking nanofibrils using the self-assembling peptide (SAP) Fmoc-FRGDF. Hesperidin was delivered using this coassembled biocompatible tissue-specific hydrogel, which was made of fucoidan laced within a self-assembling peptide backbone. The coassembly of the SAP with fucoidan was reported to improve the mechanical properties (from 9.54 Pa of Fmoc-FRGDF hydrogel to 7735 Pa of coassembled hydrogel at 6 mg/mL fucoidan concentration), form thicker nanofibril bundles at 4 and 6 mg/mL fucoidan concentration, improve the EE of hesperidin from 72.86% of Fmoc-FRGDF hydrogel to over 90% of the coassembled hydrogels, and demonstrate the efficiently controlled release of hesperidin in vitro [22]. However, the physical cross-linking sites in nanogels are unstable because they rely on weak interactions between polymer chains, such as hydrophobic or electrostatic interactions or hydrogen bonds. Relatively weak noncovalent interactions mean that it is generally difficult to obtain physically cross-linked and size-controlled stable nanogels. In addition, after injection into bodily fluids, they will be very diluted and dissociated into hydrophilic polymers, which can lead to the premature release of therapeutic agents, causing unwanted side effects. The physical self-assembly of preformed polymers (or monomers) followed by chemical cross-linking, on the other hand, is a promising method for producing stable nanogels without the use of surfactant or solvent [5,23]. Cross-linking plays an important role in improving the drug release time, targeted delivery systems, drug bioavailability, and, most importantly, cytotoxicity. Sana et al. used a dispersion polymerization technique to create a poly (acrylamide-co-diallyldimethylammonium chloride) nanogel (PAD-NG) with the free radical initiator potassium persulphate (KPS), potassium hydrogen sulphate (KHSO_4_) as a co-initiator, and N, N-methylene bisacrylamide (NN-MBA) as a chemical cross-linker. Figure 5 depicts a model cross-linked poly (Am-co-DADMAC) copolymer structure. As the percentage (%) of NNMBA, a chemical cross-linker, increased from 0.05 to 0.15%, the percentage of loading decreased, resulting in a drop in the percentage EE of the NGs from 76.34 to 41.73%. It is clear that as the cross-linking density increased, the polymer chain mobility decreased for the encapsulation of the 5-FU molecules, resulting in a decrease in the EE. The percentages of DL and % EE values increased when the amount of 5-FU increased from 0.1 to 0.3% at a constant amount of NN-MBA 0.1%. The nanogels were used as an intracellular drug delivery vehicle, encapsulating 5-fluorouracil (5-FU) with 76.34% efficiency [1].

This chemical cross-linking/physical self-assembly is particularly suitable for the production of stimuli-responsive biodegradable nanogels made from biopolymers. However, the method’s low efficiency must be addressed before it can be fully utilized for drug delivery. An alternative to chemically activated cross-linking is photo- or radiation-induced cross-linking, which was developed to stabilize polymer assemblies. In comparison to other chemical methods, radiation-based nanogel synthesis has significant advantages because it does not require the use of potentially harmful or toxic cross-linking agents, initiators or catalysts, and other additives. Polymer and water are typically the only substrates. That is why the method is so appealing for biomedical applications [24]. In radiation-induced synthesis, the reaction can be initiated by producing free radicals in a very simple system—an aqueous solution of a hydrophilic polymer. When a polymer is exposed to ionizing radiation (typically gamma rays or high-energy electrons) in a dilute aqueous solution, water absorbs the majority of the radiation energy. As a result, reactive species with short lifetimes such as OH radicals, H-atoms, and hydrated electrons are formed. Hydroxyl radicals and hydrogen atoms can rapidly extract hydrogen atoms from polymer chains, resulting in the formation of polymer radicals and the intramolecular cross-linking of linear polymers [25]. Matusiak et al., for example, used radiation to create poly(acrylic acid)—PAA-nanogels and microgels. In this method, dilute, deoxygenated aqueous solutions of poly(acrylic acid) PAA were irradiated using two sources of irradiation: γ-source and electron accelerator. Because of the prevalent intermolecular cross-linking, the former method produces mostly microgels, whereas the latter promotes the intramolecular recombination of PAA-derived radicals and, as a result, the formation of nanogels [26]. The ability to control the size of nanogels is critical for any biomedical application. The control of the nanogel particle size is essential because it affects the circulation time in the blood, viscosity, and drug loading capacity. Sütekin et al. describe a simple and reproducible method for producing biocompatible nanogels of poly(N-vinyl pyrrolidone) (PVP) via electron beam (e-beam) (NGEB) or the gamma irradiation (NGG) of dilute aqueous solutions. The effects of various parameters on the sizes of nanogels were investigated, including the total absorbed dose, dose rate, polymer concentration, and molecular weight. PVP nanogels with sizes ranging from 30to 250 nm can be prepared by controlling polymer and radiation source-based parameters. This is a popular size range in biomedical applications. Thus, radiation-induced nanogel synthesis is a technique that could be applied to any radiation cross-linking type of water-soluble polymer [27].

## 4. Inherent Properties of Nanogels

### 4.1. Biocompatibility and Degradability

Nanogels are said to be biocompatible as they work without causing harmful effects. Therefore, cytotoxicity testing is important in the field of biomaterials because it helps determine proposed biomedical applications.Biocompatibility and cytotoxicity are often tested in vitro. Nanogels are generally believed to be non-cytotoxic, although this is sometimes dose- and time-dependent.The International Organization for Standardization (ISO standards) is used for the biological evaluation of a new biomaterial before clinical use. This standard states that the morphological measurements of cell damage, cell growth, and particular aspects of cell metabolism can be used to validate cytotoxicity assays. Usually, calorimetric assays such as the MTS assay (5-(3-carboxymethoxyphenyl)-2-(4,5-dimethyl-thiazoly)-3-(4-sulfophenyl) tetrazolium),inner salt assay, the MTT assays (3-(4,5-dimethylthiazol-2-yl)-2–5-diphenyltetrazolium bromide), WST-1 assays (2-(4-Iodophenyl)-3-(4-nitrophenyl)-5-(2,4-disulfo-phenyl)-2H-tetrazolium, monosodium), WST-8 assays (2-(2-methoxy- 4-nitrophenyl)-3-(4-nitrophenyl)-5-(2,4-disulfophenyl)-2H tetrazolium, monosodium salt), and LDH assays(lactate dehydrogenase)areused to evaluate the cyto-tolerance of anewly synthesized nanogel [28,29,30]. Das et al. synthesized a dextrin-and-poly-(acrylic acid)-based nanogel [n- Dxt-p(MBA)-pAA] to deliver doxorubicin hydrochloride to human osteosarcoma cancer cell lines (MG 63). The nanogel was biocompatible and nontoxic in an in vitro cytocompatibility assay against human mesenchymal stem cells (hMSCs). The encapsulation efficiency and the amount of doxorubicin found were about 86% and about 27%, respectively. The Dox-loaded nanogels’ in vitro cytotoxicity against the MG 63 cancer cells showed that the nanogels were taken up by the cancer cells and effectively killed [31].Pereira et al. studied the biocompatibility of glycol chitosan nanogel (GC-nanogel) and demonstrated that GC nanogel is a potential biocompatible vehicle for drug delivery because it does not activate the complement system, does not escape MPS, does not interact with red blood cells, and has been found not to cause thrombosis [32].In another study, Wei et al. synthesized three types of hydrophilic photoinitiator-functionalized nanogels based on polyethylene glycol dimethacrylate (PEGDMA),oligo(ethylene glycol) monomethyl ether methacrylate (OEOMA), and 2-hydroxy-4′-(2-hydroxyethoxy)-2-methylpropiophenone. The cytotoxicity of these nanogels was evaluated on HeLa cells.The viability of the HeLa cells was determined using the MTT cytotoxicity assay method and this showed that the nanogels have outstanding biocompatibility [33].To reduce the growth of breast cancer cell lines, Guo et al. designed a nanogel based on positively charged chitosan (CS).They used the model drug 10-hydroxycamptothecin (HCPT), which was easily entrapped withinthe core to form CS/HCPT. The in vitro cytotoxicity of CS/HCPT and free HCPT was measured in 4T1 cells using a standard MTT assay.At 490 nm absorbance, MTT products were measured using a Bio-Rad 680 microplate reader. The cytotoxicity testing of these nanogels revealed the excellent biocompatibility of CS/HCPT and thus, positively charged CS-based nanogels were used as potential drug delivery systems [34].

### 4.2. Swelling Property in Aqueous Media

The swelling phenomenon is an intrinsic property of hydrogels. Due to their large surface area, nanogels have a greater swelling ability than conventional hydrogels. Nanoformulation creates a high fluid exchange capacity with the environment [35]. The swelling rate is controlled by the following key parameters such as the nanogel morphology, cross-linker hydrophilicity, length between cross-link points, and number of reactive groups per cross-link chain. Pikabea et al. synthesized poly(2-diethylaminoethyl) methacrylate-based nanogels using two bifunctional cross-linkers (based on ethylene glycol:EGDMA and PEGDA, containing methacrylate and acrylate groups, respectively) and a multifunctional agent (dextran-based cross-linker:Dex40MA86, contains 86 methacrylate groups).It was found that the highest swelling was in the PEGDA cross-linked nanogels due to their higher hydrophilicity, while the swelling ability of the nanogels cross-linked with multifunctional Dex40MA86 was low compared to the other types of nanogels. This is because the multifunctionality of Dex40MA86 limits the movement of the sub-chains, and swelling becomes more difficult [36]. In addition, the swelling rate of nanogels is also dependent on endogenous (e.g., enzymes, pH, reduction, etc.) or exogenous stimuli (e.g., temperature, light, and magnetic fields). For example, a cross-linked poly(acrylic acid)-(pAA) nanogel was synthesized and the swelling ability of the nanogel with pH was tested using the DLS technique. Nanogels exhibit very high swelling changes with pH. It was observed that the largest changes in the nanogel diameter occur at pH between 4 and 7. For example, the hydrodynamic diameter of the nanogel increased from 122 nm (at pH 2) to 1990 nm (at pH 8) [37].

Tamura et al. described the pH-dependent swelling behavior of PEGylated nanogels in a study, and then demonstrated the dependence of pH-sensitive swelling on the density of PEGylated nanogel cross-linking. Dynamic light scattering (DLS) was used to investigate the pH-dependent changes in the hydrodynamic radius (Rh) of nanogel particles. The Rh value of the sample varied significantly around the pKa value, and the size of the PEGylated nanogels increased at pH levels lower than the pKa value. They reported that at lower pH, the nanogel size decreased and depended on the cross-linking density of the core. The cause of this swelling phenomenon is the protonation of the nucleus. This swelling phenomenon plays an important role in the release of pH-sensitive drugs [38]. Daniel et al. prepared cross-linked κ-carrageenan nanogels and reported that the swelling ability of the nanogels was found to be thermoresponsive within the temperature range acceptable for living cells (37–45 °C). Differential scanning calorimetry (DSC) was used to characterize the thermal behavior of the nanogels, and the DLS technique was used to measure the mean hydrodynamic diameter (Rh) of the nanogels. It was observed that the size of the κ-carrageenan nanogels increased as the temperature increased from 25 to 45 °C. In addition, the average size of the swollen κ-carrageenan nanogels was also observed to increase with increasing κ-carrageenan content (Rh value increased from 344 nm to 475 nm at 25°C for 1.0 and 4.0% by weight of κ-carrageenan), this may be due to the larger number of biopolymer chains available inside the swellable nanosphere. Therefore, temperature-sensitive nanogels are promising materials in the development of smart temperature-sensitive drugs [39].

### 4.3. Higher Drug Loading Capacity and Drug Release

The inclusion of drug molecules into nanogels is an important factor for any drug delivery system, and the higher the loading capacity of the drug, the lower the nanocarrier used for the drug. Because of their swelling ability, nanogels have a high drug loading capacity and encapsulation efficiency. Like all other nanoparticles, drug loading occurs by(i) physical entrapment: this can refer to hydrophilic–hydrophilic, hydrophobic–hydrophobic, and/or hydrogen bonds or Van der Waals or electrostatic interactions; or(ii) the covalent binding of biologically active molecules; or (iii) controlled self-assembly (which can be controlled by numerous stimuli such as pH, temperature, light, ion exchange, etc.). Other factors can also influence the load-carrying capacity, such as the composition, nanogel particle size, molecular weight, hydrogel, and different functional groups of the hydrogel unit. Moreover, drug release from nanogels is affected by a variety of factors, including the type of drug, its interaction with the nanogel, the nanogel’s structure, and environmental conditions [40,41]. For nanogels, the drug release mechanism can be classified as follows: (i) a diffusion control mechanism, (ii) a swelling control mechanism, and (iii) a chemical control mechanism. The drug release process depends on the physicochemical properties of the nanogel and how the drug is loaded into the nanogel. To allow the drug to escape from the nanogel, the nanogel expands the size of the network by swelling and opening the door for drug diffusion. Therefore, the release properties mainly depend on the network size in the nanogel matrix. Thus, small drug molecules can diffuse easily compared to macromolecules such as proteins, oligonucleotides, peptides, etc. The swelling of nanogels and thus drug dissociation can be triggered by physical or chemical stimuli such as temperature, pressure, pH, magnetic field, light, ions, and specific molecular recognition [42,43].

### 4.4. Colloidal Stability

The colloidal stability is the essential physicochemical property of nanoparticles and is predetermined by their structural and functional design (core and coating materials). The colloidal stability is greatly influenced by environmental factors such as pH, salt, temperature, proteins, cells, and so on. Because the coating material is the first part of the particle to come into contact with the environment, it determines the colloidal stability behavior. The coating material thus provides immediate biocompatibility and colloidal stability throughout its lifetime. It has been successfully demonstrated that charged polysaccharides, such as alginate, chitosan, hyaluronic acid, or charged dextran derivatives, can improve colloidal stability in biological media and sustain it for extended periods of time. Proteins are other biopolymers that are gaining importance in colloidal stability and nanomedicine. In the field of nanomedicine, polyethylene glycol (PEG) or its analogues, e.g., POEGMA, are the most commonly used polymers for coating colloidal particles. This is because the strong hydration layer of these nonionic polymer coatings is responsible for the NPs’ strong colloidal stability at an elevated ionic strength [44,45].

### 4.5. Non-Immunologic Response

Nanogels display remarkable physical, chemical, electrical, and biological characteristics [46]. In addition to their maximum therapeutic efficacy with minimal side effects and desired site-specific release behavior, nanogels as drug delivery systems show minimal toxicity, non-immunogenic behavior or negligible immunogenic response, and satisfactory biocompatibility and biodegradability with non-toxic degradation products to enhance their targeting effectiveness in cancer therapy [47,48]. Presently, most nanogels are limited to their utilization in pre-clinical laboratory testing and must be explored more efficiently for in vivo trials towards next-generation clinical translation for precision and personalized medicine [46].

## 5. Biomedical Applications of Stimuli-Responsive Nanogels Systems

**Figure 6:**
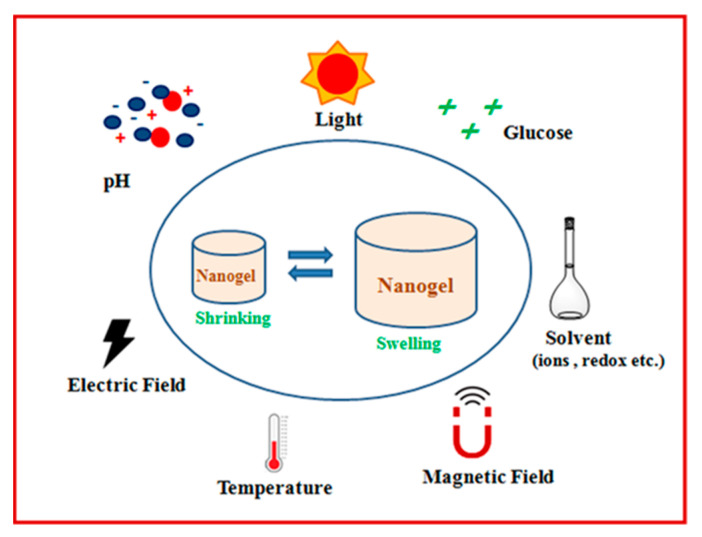
Schematic representation of stimuli-responsive nanogels.

Stimulus-sensitive nanogels respond dramatically to very small changes in environmental stimuli including enzymes, light, temperature, ionic strength, electric and magnetic fields, and pH (Figure 6).

A vast array of stimuli-responsive nanogels have been developed recently for use in drug delivery applications that make use of one or more environmental stimuli [6,7].

### 5.1. Single Stimulus-Responsive Systems

#### 5.1.1. pH-Responsive Nanogels

**Figure 7:**
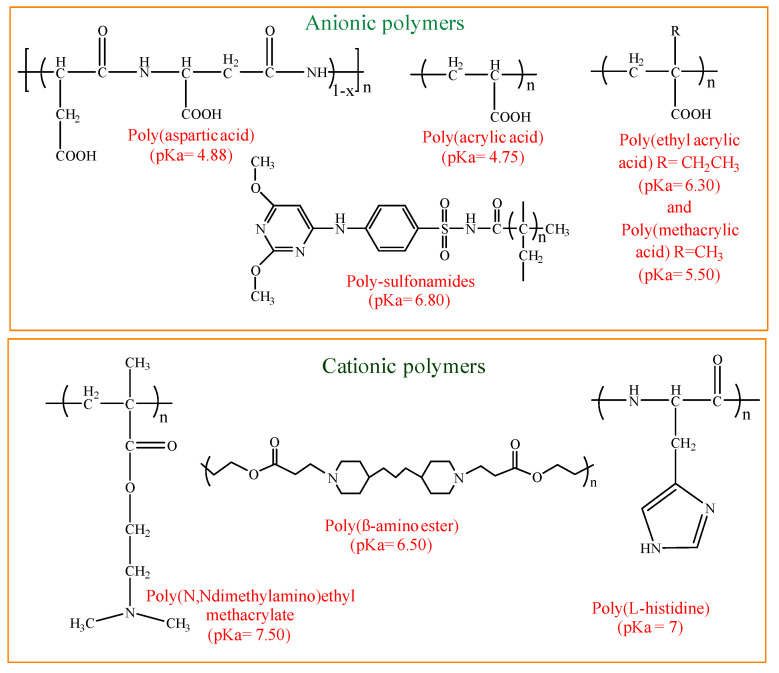
Chemical structure of some important pH-sensitive cationic and anionic polymers and their pKa values.

Among various stimuli, pH is one of the most commonly used stimuli, and the pH-responsive properties of nanogels could be crucial for drug loading as well as release. The pH of healthy tissue is 7.4, while the pH of tumor tissue ranges from 6.5 to 7.0, and the pH of the stomach ranges from 1.0 to 3.0. Therefore, pH-responsive nanogels can take advantage of the variations in pH in various body parts to deliver targeted drugs. Therefore, pH-responsive nanogels enhance drug release and minimize drug loss to off-target sites. pH-responsive nanogels are typically prepared by incorporating acidic or basic functional groups into the polymer back bone [8]. These groups accept or release protons when the pH of the external environment changes. Figure 7 summarizes the various protonatable pH-sensitive polymers and their pKa values [15].

Malaria and cancer are considered deadly diseases worldwide. Recently, to hit two targets with just one arrow, Rashidzadeh et al. have created pH-sensitive nanogels to treat malaria and cancer. They synthesized a pH-responsive nanogel (PMAA-BSA) using bovine serum albumin and methacrylic acid via the distillation precipitation polymerization (DPP) technique for smart chloroquine (CQ) delivery. By encapsulating BSA on the PMAA nanogel surface, its plasma half-life and colloidal stability were enhanced while its potential systemic toxicity was significantly reduced. These nanogels showed a high drug loading efficiency (26.42%) and CQ loss was minimized during its circulation in the blood. Upon targeted and site-specific delivery, these nanogels were able to rapidly release high doses (92.03%) of CQ inside tumor tissue and parasite digestive vacuoles (DVs) at low pH. More importantly, at 24 and 48 h, this pH-responsive nanogel decreased the IC50 of CQ in MCF-7 cells by about 2.8 and 1.9 times, respectively. PMAA-BSA-CQ, through its synergistic effect, demonstrated excellent antispasmodic activity as well as significant anticancer potential under in vitro and in vivo conditions. Importantly, BSA-conjugated nanogels can significantly reduce cytotoxicity, biotoxicity, and acute toxicity. Additionally, PMAA-BSA-CQ completely eliminated parasites in mice infected with Plasmodium berghei and prolonged their survival rate [49]. A pH-responsive polyvinylpyrrolidone (PVP)-based nanogel was developed using γ radiation for the controlled release of 5-fluorouracil (5-FU) by Naranjo et al. The pH, PVP percentage, and dose of radiation all had a significant impact on drug loading and release from nanogels. Docking simulations helped explain the influence of pH environment on drug loading; the 5-FU–NG interaction was validated and demonstrated that the encapsulation efficiency = 17% and drug loading = 83% at low pH. The release pattern responded to pH changes over a significant physiological range from 1.2 to 7.4, and the release rate increased under acidic conditions (87% NG in 72 h). These characteristics suggest that PVP nanogels may be used to treat colorectal cancer by serving as nanocarriers for anticancer medications like 5-FU [50]. Jayakumar et al. prepared biodegradable and biocompatible doxorubicin-loaded chitin nanogels for anticancer drug delivery applications. Drug release studies have shown that a 32% release was observed in the first hour at both neutral and acidic pH. Within 24 h, 60% of the drug was released in an acidic environment and 40% in a neutral pH. Consequently, the doxorubicin-loaded chitin nanogels showed controlled, pH-dependent doxorubicin release [51].

Using formaldehyde (FDNG) and glyoxal (GDNG) as cross-linking agents, Manchun et al. created a pH-responsive dextrin nanogel for the targeted delivery of doxorubicin (DOX) to colorectal cancer. The release profiles demonstrated that a higher DOX release occurred at pH 5. (endosomal pH) and pH 6.8 (tumor tissue condition), where both DNGs demonstrated pH-dependent drug release properties. The results of the cellular uptake indicate that DOX-loaded FDNG shows promise as a possible drug delivery vehicle for the treatment of colorectal cancer [52]. Sahu et al. fabricated chitosan-based pH-responsive biodegradable nanogels (FCNGLs) loaded with 5-fluorouracil (5-FU) for the effective treatment of melanoma. FCNGL nanogels exhibited pH-dependent sustained 5-FU release and important medicinal possibilities against melanoma even when using very little medication (0.2% *w*/*v*). The cumulative drug release of FCNGL exhibited sustained 5-FU release behavior and followed Higuchi’s kinetic model, which revealed the pH-sensitive behavior and site-specific physiological properties of drug release that mimic melanoma conditions. FCNGL has almost no blood hemolytic properties and is therefore considered a secure medication for in vivo administration. The anticancer efficacy of locally applied low-dose FCNGL (0.2% 5-FU *w*/*v*) is significantly higher against chemically induced melanoma animal tumor models [53]. Yang et al. prepared HA-mPEG diet nanogels encapsulated with the protein drug cytochrome c (CC) for dual-targeted protein delivery to CD44-overexpressing MCF-7 cells. According to their findings, HA-mPEG diet nanogels are excellent choices for promoting cellular internalization, sustaining circulation in the blood over an extended period of time, and controlling pH-dependent protein release. Significantly faster CC release was observed from HA-mPEG-Diet nanogels, exhibiting 51.2% and 92.6% protein release in 24 h at pH 6.5 and 6.0, respectively. This rapid release of CC is brought on by the benzoic imine bond’s pH-induced cleavage (pH < 6.5). Higher anticancer activity was obtained by incorporating pH-sensitive dynamic benzoic imine bonds into HA-mPEG-Diet nanogels, which greatly enhanced cellular internalization and induced CC release in either CD44-positive MCF-7 cells or CD44-negative HeGp2 cells [54].

Dopamine-grafted hyaluronate nanogels for bortezomib (BTZ), a hydrophobic anticancer medication and proteasome inhibitor, were obtained by Liu et al. They discovered that due to the presence of catechol groups, BTZ was more efficiently captured in the nanogels, and the drug loading increased from less than 1% to 8.5% by modifying the nanogels with 29% dopamine. Because dopamine groups are present on the nanogel scaffold, the release of BTZ was pH-controlled. To demonstrate that catechol-containing nanogels are pH-responsive, in vitro drug release studies were performed and the physiological pH conditions were acidic (pH = 5), which was beneficial for improving therapeutic efficiency and reducing side effects. In vivo antitumor experiments showed that loading bortezomib (BTZ) into nanogels significantly improved the therapeutic efficacy with a 2-fold decrease in the tumor volume over 14 days of treatment compared to free BTZ [55]. In this order, Wang et al. also prepared acid-sensitive PEGOE-OMAP nanogels loaded with DOX for drug delivery in tumor therapy. The prepared nanogels showed good stability in a physiological environment, while they showed a pH-controlled drug release profile in an acidic environment. At pH 5.0, it was discovered that the cumulative doxorubicin (DOX) release rate of the PEGOE-OMAP/DOX nanogels reached 83% in 48 h. Surprisingly, at pH 7.4 and 6.8, the DOX-loaded nanogels showed less cytotoxicity than free DOX; this could be because of the controlled drug release. According to in vitro experiments on cell uptake and cytotoxicity, the prepared nanogels showed the greatest levels of drug accumulation and cytotoxicity when applied to EMT6 cells. Furthermore, compared to the other three drug-loaded nanogels, the PEGOE-OMAP/DOX nanogels were found to significantly inhibit EMT6 tumor growth in vivo, with a TGI of 79.24%. Consequently, the in vivo distribution of DOX demonstrated the great potential of the PEGOE-OMAP nanogels as drug carriers in cancer therapy [56]. Hepatic carcinoma (HCC) is one of the most common types of cancer, and treating it has proven to be a therapeutic challenge. Doxorubicin (Dox) is a major chemotherapeutic agent used in the treatment of liver cancer. Arunraj et al. created a pH-sensitive intratumoral Dox–chitin–PLA CNGs nanogel system for liver cancer in this context. The control composite nanogels and their drug-loaded counterparts had size ranges of around 90 and 270 nm, respectively. With an entrapment efficiency of 86%, dox was effectively loaded onto the chitin–PLA CNGs. At acidic pH, the Dox–chitin–PLA CNGs showed increased swelling and drug release. The Dox–chitin–PLA CNGs were tested for cytotoxicity against HepG2 (human liver cancer) cell lines and found to have a significant cytotoxic effect. As a result, the Dox–chitin–PLA CNGs system could be a promising drug delivery system for the treatment of liver cancer [57].

#### 5.1.2. Temperature-Responsive Nanogels

Like pH, temperature is also an important and unique stimulus that can be easily applied. Temperature-responsive nanogels exhibit a contraction–swelling behavior triggered by ambient temperature, which facilitates the control of drug release from the nanogels. The temperature-dependent properties of nanogel systems result in phase transitions occurring above or below a certain temperature. Temperature-responsive hydrogels are generally classified into two types depending on their temperature behavior. The first group has a lower critical solution temperature (LCST) and the second group has the upper critical solution temperature (UCST). If heating reduces the solubility of the hydrogels, they are characterized according to the LCST. Above the LCST, these hydrogels become insoluble and the formulated nanogels shrink, whereas below the LCST, the nanogels swell. However, if the hydrogels become more soluble upon heating, they can be characterized according to UCST. Below the UCST, these hydrogels become insoluble and the formed nanogels shrink, whereas above the UCST, the nanogels swell [58].

**Figure 8:**
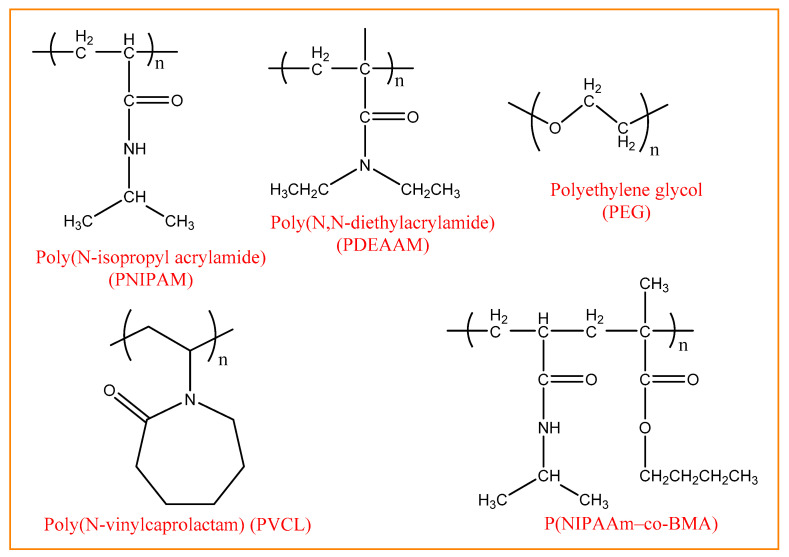
Structures of some temperature-sensitive polymers.

Thermoresponsive nanogels are commonly synthesized from polymers with amide or vinyl ether groups. Figure 8 depicts the structure of some of these polymers.

Poly (N-isopropyl acrylamide) (PNIPAM), poly (N-isopropyl methacrylamide) (PNIPMAM), poly(N,N-diethylacrylamide) (PDEAAM), and poly(N-vinylcaprolactam) (PVCL) are thermally responsive polymers with amide group. Polyethylene glycol (PEG) is a major polymer with polyether groups. Polymers with vinyl ether groups are poly(2-[2-methoxyethoxy] ethyl methacrylate) (PMEO2MA) and oligo (ethylene glycol) methyl ether methacrylate (OEGMA). Cholesterol, poly(L-lactide) (PLLA), poly (lactide-glycolic acid) (PLGA), and PCL are important hydrophilic polymers with hydrophobic groups. Generally, the thermoresponsive polymers described above are conjugated to polysaccharides to improve biocompatibility and other desirable properties. As an alternative approach to forming thermosensitive nanogels, hydrophobic substances like cholesterol and PLLA can be mixed with hydrophilic polymers. One of the most researched thermosensitive polymers, NIPAM, is frequently utilized to create nanogels for use in biomedical applications [59]. For example, recently, nanostructured hydrogels were prepared by the radical polymerization of activated poly(N-isopropylacrylamide) (PNIPAM) nanogels with N-acryloylglycinamide (NAGA) as nanocross-linking centers. The nanostructured hydrogels possessed a high mechanical strength, elasticity, and excellent LCST- and UCST-type swelling properties. The optical and mechanical properties of PNIPAM nanogels can be adjusted based on their concentration and degree of unsaturation in the pregel solution. The rheological behavior of the synthesized hydrogels follows dual responsive UCST and LCST behavior. These hydrogels with nanostructures show promise as materials for tissue engineering applications or as temperature sensing devices [60]. In one example, Zhou et al. synthesized temperature-sensitive nanogels using poly(N-isopropylacrylamide)-acrylic acid copolymer (NIPAM-co-AA). To obtain smart thermosensitive radiopaque nanogels (INCA), 200 mg/mL of iohexol (radiopaque contrast agent) was composed together with 7 wt% NIPAM-co-AA nanogels. The aqueous gel dispersion’s good dispersibility and small particle size allowed for the complete embolization deep within peripheral vessels, resulting in good fluidity. Furthermore, NIPAM-co-AA nanogels induced gelation at about 34 °C because of their excellent temperature sensitivity. Additionally, it was verified that INCA nanogel could be applied more successfully to the distal portion of a rabbit right renal artery in a normal renal embolization model. The rabbit’s right kidney displayed considerable ischemia necrosis, atrophy, and calcification after 42 days of embolization, suggesting that INCA nanogels can accomplish total renal artery embolization. As a result, there is hope for this biocompatible nanogel embolic material to become a novel medical embolic material in clinical settings [61].

Lee et al. developed thermosensitive poly(N-isopropylacrylamide) (PNIPAM)-based nanogels (30–50 nm) containing timolol maleate (TM) and fabricated bicontinuousmicroemulsion contact lenses (BMCLs). Nanoporous BMCLs containing thermosensitive TM-loaded nanogels could effectively release TM at body temperature. In aqueous media, it was observed that regardless of temperature, the initial timolol release from BMCLs directly soaked with the drug was achieved at approximately 72–94%, followed by the sustained release of the remaining drug within 24 h [62]. Topotecan (TCN)-loaded thermosensitive nanocargo (TCN-TS-NC) with improved antitumor activity was created by Zhang et al. for intramuscular (IM) delivery. To create TCN-TS-NCs in this context, temperature-responsive solid lipid nanoparticles (SLNs) loaded with TCN were added to a poloxamer solution that was temperature-sensitive. The lower amount of TCN released from the TCN-TS-NCs compared to the TCN-SLNs, TCN solution, and TCN-emulgel indicates the better control of drug release of the TCN-TS-NCs. A significant improvement in antitumor activity was observed in tumor-bearing athymic nude mice treated with TCN-TS-NCs compared to control mice treated with TCN solution and TCN-emulgel [63]. Sahle et al. synthesized thermoresponsive nanogels based on dendritic polyglycerol-N-isopropylacrylamide (dPG-NIPAm) for the controlled delivery of drugs through hair follicles. These nanogels exhibited cloud point temperature (Tcp) values of 32–37 °C, which are ideal for skin application. They studied ex vivo the temperature-dependent release of the dye coumarin 6 loaded as a model drug from thermoresponsive nanogels in hair follicles and found that the dye release was significantly increased over Tcp in the nanogel [64].

Li et al. designed and fabricated size-controlled, temperature-sensitive, curcumin (Cur)-loaded PEDOT@PNIPAAm nanogels for applications in removing reactive oxygen species and killing pathogenic bacteria. Using modified precipitation polymerization, poly(N-isopropylacrylamide) was used to create nanogels that encapsulated curcumin and poly(3,4-ethylenedioxythiophene) nanoparticles. Nanogels containing curcumin have the ability to scavenge reactive oxygen species and inactivate harmful bacteria. PEDOT@PNIPAAm nanogels exhibited a 76.7% encapsulation efficiency and 19.1% loading capacity, indicating that PEDOT@PNIPAAm possesses excellent drug delivery properties. Fluorescence spectra obtained under NIR laser irradiation were used to measure the amount of Cur released from the nanogels, and the Cur-loaded nanogels were found to have a high photothermal conversion efficiency (56.8%). Furthermore, the fluorescence spectra showed that the intensity of Cur gradually decreased with an increasing concentration of nanogels, indicating that the PEDOT@PNIPAAm-Cur nanogels were temperature sensitive [65].

Zavgorodnya et al. developed a temperature-sensitive poly(N-vinylcaprolactam) nanoparticle (νPVCL) nanogel film for the delivery of multiple drugs. They demonstrated the temperature-triggered release of diclofenac sodium (a nonsteroidal anti-inflammatory drug used to treat osteoarthritis pain) from a multilayer hydrogel (νPVCL) in solution through a synthetic skin membrane. Following a 24 h period, the total quantity of diclofenac that was transported from the (νPVCL) hydrogel at 32 °C (average surface temperature of human skin) was 12 times higher than that at 22 °C. Thus, they illustrated how (νPVCL) multilayer hydrogels might be applied to the delivery of multiple drugs [66]. Wang et al. developed a thermoresponsive nanogel based on chitosan (CTS) and acrylamide (AAm) blend CTS-poly (NIPAAm-co-AAm5.5) copolymer with N-isopropylacrylamide (NIPAAm) for paclitaxel (PTX) delivery. With a loading efficiency of 9.06±0.195%, this nanogel can load PTX. The drug is released in a temperature-dependent manner, with a significantly faster rate at higher temperatures than at the volume phase transition temperature (VPTT). Thus, these nanogels are especially appealing for the combination of anticancer medications and hyperthermia with heat-triggered drug release [67].

#### 5.1.3. Glutathione (GSH)-Responsive Nanogels

The “bioreduction” stimulus arises from the electrochemical response of specific redox-reactive functional groups that experience differences in oxidation states. The main components in the production of redox-responsive nanogels are cross-linking agents. They have disulfide bonds (-S-S-), diselenide bonds (-Se-Se-), and ditellurium bonds (-Te-Te-) as functional redox-active units to retain therapeutic agents. When a redox trigger occurs, it degrades and breaks down to release its payload. Generally, these bonds are broken into their respective reduced forms when reducing agents such as glutathione (GSH) and dithiothreitol (DTT) are present, ensuring biodegradability and rapid drug release [10]. Many redox-sensitive disulfide-containing linkers have been developed for use in drug delivery carriers. But, due to the extremely high sensitivity of the diselenide bond (-Se-Se-), the responsiveness of the Se bond to low-concentration redox environments can be exploited in the form of smart drug delivery systems [68]. For example, Yu et al. introduced the selenylsulfide bond (Se-S) as a mild reduction-responsive bond. They prepared magnetic DOX-loaded Se-S-alginate nanogels (MDSeSAN gels) to achieve reduction-triggered release. Therefore, the nanogels exhibited mild reductive responsive targeted release, as Se-S had a higher reductive cleavage than S-S or lower than Se-Se [69]. Zhao et al. developed the system “Apt-GS/siRNA” by combining gelatin-based nanogels with nucleolin-targeting AS1411 aptamer and deoxynucleotide-substituted siRNA via a disulfide linker (-S-S-) to generate transient small interfering RNA (siRNA) to achieve docking. Under reducing conditions, these Apt-GS/siRNA nanogels exhibited desirable siRNA release via disulfide cleavage. Moreover, Apt-GS nanogels with these disulfides showed good biocompatibility in vitro and were able to protect the cargo and facilitated siRNA release through disulfide cleavage upon the addition of DTT [70]. Recently, through the emulsion polymerization of HSEMA and R848 prodrug (R848-HSEMA), Wang et al. created a redox-responsive nanogel delivery system (R848 gel). GSH-induced R848 release was observed compared to pure phosphate-buffered saline (PBS solution, 0.01 mol/L). The in vitro experiments show that R848 gel is non-toxic and can effectively activate BMDCs and BMDMs. The in vivo studies showed that R848 gel exhibited stronger antitumor effects compared to the free drug and no dramatic changes in body weight. The analysis of intratumoral immune cells after treatment showed that R848 gel helps activate the immunosuppressive tumor microenvironment (TIME). Therefore, R848 gel is a simple but effective R848 vehicle to improve cancer immunotherapy [71].

Degirmenci et al. utilized the self-assembly of dextran polymers coupled to β-cyclodextrin (βCD) and a disulfide group-containing bisadamantine (Ada) cross-linker through host–guest interactions in aqueous media to achieve redox responsiveness in nanogels.Prepared nanogels co-loaded with an adamantane-containing cyclic peptide-based cell-targeting device and the anticancer drug doxorubicin (DOX). The use of disulfide-containing bisadamantane-based cross-linkers causes the redox-reactive degradation of the nanogels upon exposure to glutathione (GSH). The treatment of MDA-MB-231 breast cancer cells with non-targeted and targeted βCD nanogels resulted in increased internalization by targeted RGD moieties. Blank nanogels showed no cytotoxicity, whereas the targeted βCD nanogels showed a higher cytotoxicity toward GSH-rich MDA-MB-231 breast cancer cells than normal cancer cells [72]. Ma et al. designed a tumor microenvironment-responsive nanogel (termed DPH NG) using the reductive cross-linking of purpurin 18 (P18) and 10-hydroxycamplothecin (HCPT). It showed high drug loading, controlled drug release, and deep tumor perforation. P18 is a potent near-infrared fluorescence (NIRF)/magnetic resonance (MR) imaging feature in this context. They reported that DPH-NG could selectively accumulate at the tumor site through the EPR effect and had excellent tumor permeability due to its nanosize. The disulfide bond of DPH-NG was activated by the high concentration of glutathione (GSH) in the tumor, resulting in the release of HeP18 and HCPT. As a result, when exposed to 660 nm laser radiation, the released drug displays both the phototoxicity of P18 and the chemotherapeutic effect of HCPT [73].

#### 5.1.4. Biomolecule-Responsive Nanogels

All living things contain biomolecules, which are substances that react to their surroundings. Examples of these include glucose, proteins, enzymes, and nucleic acids. Biomolecule-responsive nanogels have garnered significant interest among the different types of endogenous stimuli-responsive nanogels that have attracted great attention. Biomolecule-responsive nanogels can undergo structural changes in response to biomolecules.

##### Glucose-Responsive Nanogels

In diabetes, the body is unable to regulate blood sugar concentrations within normal physiological values. Therefore, glucose-sensitive drug delivery systems have attracted great interest in recent years. Three main categories of glucose-sensitive drug delivery systems exist, which are based on concanavalin A (Con A), glucose oxidase (GOD), and phenylboronic acid (PBA). Natural glucose-based systems, such as glucose and Con A, are less stable due to changing environmental conditions. Therefore, synthetic glucose-based materials, namely PBA-responsive hydrogels with excellent stability properties, have been used. However, there are several obstacles to the clinical use of PBA-responsive hydrogels, including: biodegradability, low glucose selectivity, and slow reaction rates. These problems can be overcome by the rational design of hydrogels and modification of PBA [74,75]. Guo et al., for instance, proposed a sugar-responsive nanogel, p(AAPBA-AGA-BODIPYMA), that contains boron dipyrromethene (BODIPYMA) as a fluorescent donor molecule, 2-(acrylamido) glucopyranose (AGA) as the biocompatible moiety, and 3-acrylamidophenylboronic acid (AAPBA) as the glucose-sensitive moiety. Insulin was efficiently loaded with an EE and LC up to 64% and 8.2%, respectively, by the generated nanogels. Additionally, the nanogels showed signs of glucose sensitivity by swelling to a larger size when exposed to higher glucose concentrations. Additionally, it has been observed that the model drug insulin can be encapsulated in nanogels at loading levels of up to 8.2%. The release of the drug was found to be influenced by the concentration of glucose in the release medium as well as the amount of AAPBA units present in the nanogels. The cytotoxicity studies of the nanogels showed that the nanogels had good biocompatibility. Therefore, it has been suggested that such glucose-responsive nanogels have potential as a self-regulating insulin delivery system in the biomedical field [76]. A nanogel was created by the one-pot thiol-ene copolymerization of N-acryloyl-3-aminophenylboronic acid, poly (ethylene glycol) diacrylate, pentaerythritol tetra (3-mercaptopropionate), and methoxyl poly (ethylene glycol) acrylate. The synthesized nanogel contained glucose-sensitive PBA (phenylboronic acid) moieties, which was confirmed by FTIR, ICPMS, and fluorescence techniques. These glucose-sensitive nanogels were loaded with insulin and Alizarin Red S (ARS). The in vitro release studies revealed that the presence of glucose stimulated the release of ARS from the nanogels. Therefore, by increasing the glucose concentration in PBS, conditions for more potent drug release with faster release rate were achieved. Moreover, the MTT, LDH, and hemolysis tests in vitro showed that the nanogels were biocompatible and nontoxic. Therefore, glucose-induced nanogels incorporated using PBA may have great potential for self-regulated drug delivery [77]. Zhao et al. showed that glucose-responsive GOX polymer nanogels modulate H_2_O_2_ production for melanoma starvation and oxidation therapy. In vitro, these nanogels demonstrated glucose-responsive H_2_O_2_-generation activity, improved thermostability, and significantly increased GOX antitumor activity [78].

Volpatti et al. developed glucose-responsive acetalized dextran polymer nanoparticles encapsulated with insulin and found that nanoparticles synthesized from dextran with a high content of acyclic acetals (94% of residues) exhibit fast release rates compared to the cyclic acetal content (71% of residues). The in vivo analysis in both streptozotocin-induced type 1 diabetic patients and a healthy mouse model demonstrated that this delivery system was able to respond to glucose in a therapeutically relevant time frame. The glucose response of this material in animals was also confirmed, as the amount of human insulin in mouse serum increases significantly with increasing glucose levels. They also showed that in a diabetic mouse model, these co-formulated NPs were able to reduce the rise in blood glucose levels in a time frame compared to pure insulin and improved glycemic control compared to free insulin. These co-formulated NPs at 5 IU/kg also reduce the risk of hypoglycemia. Therefore, this type of glucose-responsive nanoparticle could become a common approach for the improvement of glucose-responsive insulin delivery systems [79].

##### Protein/Enzyme-Responsive Nanogels

Protein-responsive hydrogels are classified into two types: enzyme-responsive and antigen-responsive hydrogels. Enzyme-responsive hydrogels have a high substrate specificity and efficiency, and they can be run in mild conditions. Because synthetic hydrogels have fewer enzyme-sensitive functional groups in their chemical structures, obtaining enzyme-responsive properties is difficult. Therefore, they are formed through enzymatic phosphorylation and dephosphorylation reactions. Similarly, antigen-responsive hydrogels involve the highly selective and specific interactions of antibodies with their antigens. Due to its interesting property of detecting diseases in the human body, it has the potential to be widely studied in various biomedical fields. Sharifzadeh et al. also discussed how nucleic acid-responsive hydrogels are classified into three types: RNA-responsive, DNA-responsive, and aptamer-responsive hydrogels. Although natural nucleic acid-responsive hydrogels are biocompatible, they are not resistant to temperature or enzymatic cleavage. The development of stimuli-responsive hydrogels can account for not only the precise interaction of RNA and DNA, but also the highly selective binding of DNA sequences to aptamers [75]. Wang et al. synthesized enzyme-stimulated nanogels based on G4-PAMAM (polyamidoamine) dendrimers as nanocarriers for drug delivery. In this study, they used elastase as a reaction component because excess neutrophil elastase (NE) was detected in tumor tissue. The nanogel carrier was noncytotoxic and biocompatible, with a cytotoxicity comparable to free DOX. They showed that the nanogels had a much higher drug loading capacity and enzyme-induced doxorubicin (DOX) sustained release behavior compared to free DOX, which was burst-released in vitro [80].

Yang et al. designed enzyme-responsive photo-cross-linked nanogels (EPNGs) for CD44-targeted cytochrome c (CC) delivery via the UV-induced photodimerization of cinnamyloxy groups. The EPNGs exhibited a high loading efficiency and good stability in various biological media and achieved the sustained release of CC. The MTT results revealed that the empty EPNG was nontoxic, whereas the CC-loaded EPNG killed more CD44-positive A549 cells than CD44-negative HepG2 cells and free CC. Confocal images proved that the CC-loaded EPNG exhibited rapid cellular internalization via CD44-mediated cell adhesion and rapidly escaped from the endo/lysosomal compartment. IVIS images confirmed that the CC-loaded EPNG exhibited enhanced antitumor activity and possessed excellent stability that enabled specific tumor targeting. It was also reported that the antitumor ability of CC-loaded EPNG was better than that of the free CC and control group in vitro and in vivo. Therefore, these results confirmed that these EPNGs proved to be stable and promising nanocarriers for use in cancer therapy [81]. Starch-based nanoparticles have attracted the attention of researchers in recent years due to their small size, good biocompatibility, and environmental friendliness, as well as their potential uses in drug delivery systems. Nontoxic starch-based nanoparticles respond to pH, temperature, light, and other stimuli. Yu et al. reviewed the responsiveness, toxicity, digestibility, interactions with other components, and applications of starch-based nanoparticles such as starch nanospheres, starch micelles, starch vesicles, starch nanogels, and starch nanofibers [82]. Yu et al. developed hyaluronated starch nanogels for the delivery of docetaxel (DTX, a model antitumor drug). Cross-linking primary hydroxyl groups in polysaccharides (starch and hyaluronic acid) and epoxide groups in 1,4-butanediol diglycidyl ether (used as a cross-linking agent) were used to create these nanogels. The CD44 receptors of MCF-7 tumor cells were bound using hyaluronic acid exposed on the nanogel surface. As a result, the nanogels internalized into the MCF-7 cells via CD44 receptor-mediated endocytosis allowed for stimulated DTX release via Hyal-1 enzymes abundant in tumor cells. The enzymatic degrading of hyaluronic acid by tumor cell-specific enzymes (e.g., hyaluronidase-1) significantly accelerates docetaxel release from nanogels. As a result, the nanogels selectively inhibited MCF-7 tumor cell growth in vitro (via the CD44 receptor and the hyaluronidase-1 enzyme), indicating their therapeutic potential for efficient tumor ablation [83].

Yang et al. developed a lipase-responsive drug delivery nanoplatform (PGL-DPP-FLU-NPs) for multimodal antifungal therapy. In this platform, PGL acts as a lipase-sensitive encapsulating agent, DPP acts as a photosensitizer for PDT/PTT and FLU acts as a chemotherapy agent sensitive to ABT. PGL is biocompatible, and PGL-DPP-FLU NPs can promote FLU release and increase FLU concentration at the infection site by lipase secreted by C. albicans. The combination of the photodynamic and photothermal effects of DPP and ABT resulted in a synergistic antifungal effect for the antifungal nanoplatform. The PGL-DPP-FLU NPs demonstrated excellent antifungal activity in vivo against C. albicans wound infection [84]. Das et al. synthesized a ketone-functionalized water-soluble pullulan derivative and used it as a precursor to prepare chemically cross-linked elongated nanogels. Enzymatic hydrolysis studies showed that the developed nanogel fibers were sensitive to β-glucuronidase. The maximum rate of enzymatic hydrolysis (Vmax) was 20.3 nM/min, and the turnover number (K_cat_) was 0.51 min^−1^. They reported that an in vitro bacterial study found that 1 mg of PUAA-MUGlcU nanogel fibers could detect 10^8^–10^9^ CFU/mL of *E. coli* Mach1-T1 within 2 h [85]. Xiong et al. developed a novel lipase-sensitive polymer trilayer nanogel (TLN) for the on-demand delivery of antimicrobial agents to the sites of bacterial infection. By degrading the hydrophobic poly(-caprolactone) interlayer of polymeric trilayer nanogels, bacterial lipase was used to trigger antibiotic release. In the presence of lipase or lipase-secreting bacteria, drug release was faster than in the absence of lipase or lipase-secreting bacteria. Using Staphylococcus aureus (*S. aureus*) as a model bacterium and vancomycin as a model antimicrobial agent, they confirmed that TLN released almost all of the encapsulated vancomycin within 24 h only when *S. aureus* was present, significantly inhibiting *S. aureus* growth. In addition, nanogels also deliver drugs into bacteria-infected cells and effectively release the drugs to kill intracellular bacteria. Therefore, this technique can be generalized to the selective use of a variety of antibiotics to treat different infections caused by lipase-secreting bacteria and can be used to treat extracellular and intracellular bacterial infections with a variety of other antibiotics [86].

#### 5.1.5. Light-Responsive Nanogels

**Figure 9:**
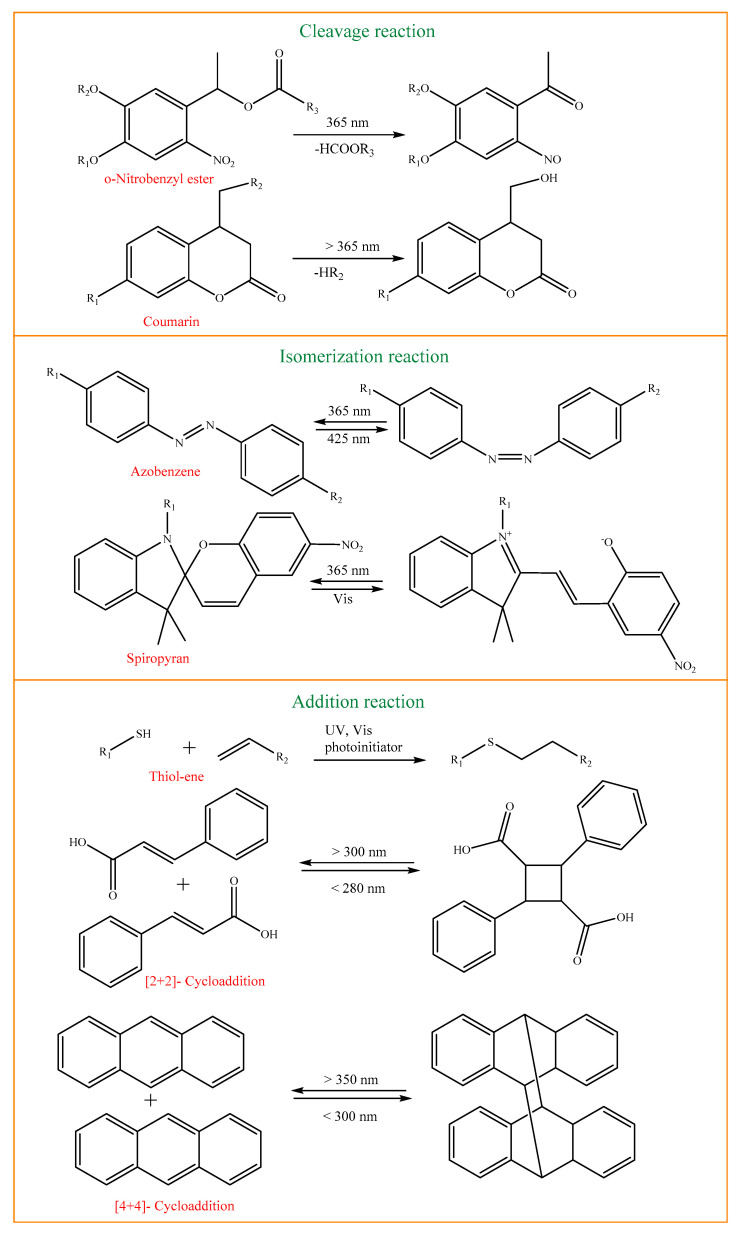
Different functional groups and photoreaction types used to design photoresponsive nanogels.

Light is an excellent external stimulus for achieving the controlled release of active molecules and there are several parameters such as wavelength and light intensity that need to be controlled to achieve the desired effect in the body. Light-sensitive nanogels are first classified into two types, followed by nanogels made from light-sensitive photoactive polymers such as azobenzene, spirobenzopyran, triphenylmethane, and cinnamonyl. The various types of photoresponsive molecules used to create photoresponsive hydrogels, as well as photoreaction types (cleavage, addition, exchange, and isomerization), are discussed below, and with representative examples shown in Figure 9 [87,88].

Light-sensitive nanogels consist of light-sensitive polymers that are capable of changing their size, shape, or ionic properties upon irradiation. When these nanogels are exposed to light, the light-responsive polymers undergo a phase transition caused by changes in the structure or polarity of the functional groups. However, because the radiation required to induce their phase transition is UV or short-wavelength visible light, which is strongly absorbed by skin and tissue and will damage tissue even at a much lower power, these light-responsive nanogels are rarely used for drug delivery. Hybrid systems composed of NPs containing noble metals such as Au and/or Ag and a temperature-responsive polymer network are another type of light-responsive nanogel. When exposed to light, the noble metal NPs absorb light and convert it to heat, causing a phase transition in the temperature-responsive polymers. In these nanogels, Ag and Au particles absorb near-infrared light that is poorly absorbed by skin and tissue. Additionally, Au NPs do not exhibit any toxicity and are therefore considered most useful for drug delivery [5,7]. For example, Augé et al. obtained NIR light-responsive polymer nanogels with a thermoresponsive polymer core displaying an upper critical solution temperature (UCST). Micellar aggregates of ABA-type acrylamide-acrylonitrile triblock copolymer are cross-linked using a nickel-bis(dithiolene) photothermal complex to absorb NIR light and efficiently convert optical energy into heat. Using an energy balance model, the photothermal conversion efficiency of the nanogels was determined, and the photothermal conversion efficiency could reach around 64%, indicating that the nickel bis(dithiolene) complex is one of the most powerful photothermal conversion agents in the NIR region around 1000 nm. Furthermore, even at a low power density of 0.16 W cm^−2^ and a low nickel bis(dithiolene) complex concentration of 61.4 g mL^−1^, the nanogel aqueous solution exhibits a significant temperature increase when exposed to near-infrared light [89].

Chen et al. prepared UV-induced degradable drug delivery nanocapsules (HA-Azo/PDADMAC) using the layering assembly of photosensitive anionic azobenzene-functionalized hyaluronic acid and cationic polydiallyldimethylammonium chloride polymer. When exposed to 365 nm light, the photoresponsive HA-azo nanocapsules undergo reversible cis–trans isomerization and the nanocapsules disintegrate from large nanocapsules into smaller polymer fragments. The nanocapsules ensure long circulation in the blood and high tumor accumulation, and also act as tumor-targeting ligands for the CD44 receptor. The authors discovered that the HA-Azo/PDADMAC nanocapsules loaded with DOX increased the cellular uptake and significantly inhibited HepG2 cell proliferation. The degradation of the UV-responsive capsule into a dispersed polymer allows for the release of the chemotherapeutically loaded drug after UV-induced dissociation and rapid removal from the tumor [90]. In another study, Hang et al. used hyaluronic acid-g-7-N,N-diethylamino-4-hydroxymethylcoumarin (HA-CM) and developed NIR- and UV-responsive degradable nanogels. Nanometer-sized HA-CM nanogels exhibit adequate doxorubicin (DOX) loading, active CD44 targeting ability, and remotely controlled intracellular DOX release upon NIR or UV irradiation. The in vitro studies reveal that the DOX-loaded HA-CM nanogels combined with NIR irradiation showed much higher efficiencies in the MCF-7 cells (CD44+) than in the U-87MG cells (CD44-) or the MCF-7 cells pretreated with free HA. These tumor-targeted photocontrolled HA-CM nanogels have great potential for cancer chemotherapy [91].

Pourjavadi et al. synthesized a nanogel based on chitosan-poly(N-isopropylacrylamide) (PNIPAM) and modified it with gold and magnetic nanoparticles to obtain a photoactivated drug carrier. This reduces the toxicity and side effects of free drugs in the body. Using the green method, gold nanoparticles (GNPs), which act as photothermal converters, were formed in situ on the surface of chitosan. It has been described that gold nanoparticles can directly destroy cancer cells through a thermal effect and can also induce drug release from heat-sensitive nanogels. When green light is irradiated onto the nanogel, the surface plasmon resonance of the GNPs generates local heat, which causes the nanogel to shrink due to PNIPAM and the release of drug molecules. This study demonstrated the achieved significant potential of the nanogels as visible-light-sensitive drug carriers and the stepwise elucidation of the drug release behavior of the nanogels [92]. Panja et al. developed a smart light-responsive and ultra-fast polymer based on branched pentaerythritol-poly(caprolactone)-b-poly (acrylic acid) (PE-PCL-b-PAA) by using iron ions (Fe^3+^) as a cross-linking agent. Nanogels are elastic in nature, which is confirmed by rheological studies, with a maximum storage modulus of 6488 Pa. The average particle size of the nanogels was tuned from 30 to 450 nm by varying both the molar concentration of Fe^3+^ and length of the polymer chain. The high zeta potential (−46 mV) of the nanogels is due to the presence of surface COOH groups, and the strong negative zeta potential promotes the remarkable colloidal stability of the nanogels and higher accumulation in cancer cells. The nanogel holds DOX drug molecules tightly and has a DLC of up to 26.2%. In the presence of laser light, the nanogels immediately undergo cross-linking and exhibit a maximum DOX release of 85.2% after only 120 min. This nanogel exhibits a very high internalization of DOX-loaded nanogels into cancer cells and acute toxicity against cancer cell lines (C6 glioma, in vitro). Histopathology confirmed that the injecting of the DOX-loaded nanogels into a C6 glioma rat model (in vivo) showed remarkable therapeutic efficacy, inhibiting tumor growth by 91% without any toxic side effects [93].

#### 5.1.6. Electric/Magnetic Field-Responsive Nanogels

One of the most common systems is magneto- and (or) electro-responsive nanogels. These systems are typically made up of two components: magneto-/electro-responsive particles and a non-magnetic/electric polymer matrix. These nanostructures are commonly referred to as hybrid nanogels or nanocomposites. These hybrid nanogels react to electric and magnetic fields by changing their properties in response to minor changes in electric current or external magnetic fields. Common magnetic/electrically responsive particles include metal particles (Fe, Co, Ni), iron oxides (Fe_2_O_3_ and Fe_3_O_4_), cobalt ferrite (CoFe_2_O_4_), nickel ferrite (NiFe_2_O_4_), carbonyl iron (CI), and carbon nanotubes (CNTs), etc. [94,95]. The magnetic NP content of each nanogel may influence its magnetic field responsiveness and the heat generated by an alternating magnetic field, which is important in the application of magnetic field-responsive nanogels in drug delivery. Salazar et al. recently created electroactive nanocomposite hydrogels (GG/PPy) by incorporating bioamine-cross-linked gellan gum (GG) networks with green-synthesized polypyrrole (PPy) nanoparticles. The anti-inflammatory drug ibuprofen (IBP) was used as a model drug and was loaded onto the GG/PPy nanocomposite hydrogels. The electric-field-driven migration of the charged IBP molecules from the neat GG hydrogel was insignificant, and the neat hydrogel had poor electrical signal sensitivity. On the other hand, electrical stimuli significantly activated the IBP release kinetics from the nanocomposite hydrogels (GG/PPy), and a pulse potential of 5 V increased the drug delivery up to 63% from the GG/PPy nanocomposite hydrogel, in contrast to the low IBP amount released in a passive form (10%). As a result of the electro-responsive behavior of the GG/PPy nanocomposite hydrogel, these materials are very appealing for controlled drug delivery triggered by electrical stimuli [96]. Pell’a et al. used emulsion polymerization techniques to create magnetic microgels based on chitosan that contained or did not contain CoFe_2_O_4_ nanoparticles. The round-shaped microgels with and without CoFe_2_O_4_ found ranged in size from (1.62 ± 0.38) μm to (1.71 ± 0.61) μm, respectively. The microgels’ release behavior was studied in the presence and absence of a magnetic field, with vitamin B12 serving as the model drug. It was reported in this study that, in the absence of a magnetic field, at pH 7.4, a fast release was observed, reaching equilibrium after 30 min. In the presence of a magnetic field, the alignment of the chains to the magnetic field promoted an initial fast release, followed by a more controlled one that lasted 50 min at pH 7.4. The presence of folic acid, which confers anti-oxidative and anti-secretory properties to the microgels, makes this type of release appealing for the treatment of gastric wounds [97]. A summary of the individual stimuli-responsive nanogels is shown in Table 1.

### 5.2. Dual Stimuli-Responsive Nanogel Systems

**Figure 10:**
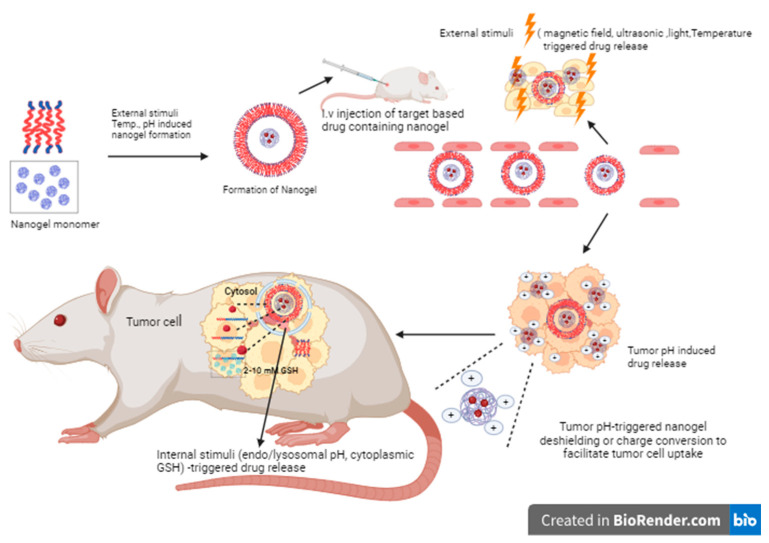
Dual- and multi-stimuli-responsive polymeric NPs as novel controlled drug delivery systems.

In recent years, the progress of dual-stimuli-responsive and multi-stimuli-responsive systems that combine multiple response functions in a single system has attracted increasing attention. The inclusion of two or more responsive moieties within the polymer increases the reactivity of nanogels. Incorporating multiple stimulation triggers into a single nanogel delivery system can increase the level of precision in the application of a desired treatment ortherapy. These two or more stimuli are combined as follows: (i) The application of external stimuli such as pH and temperature facilitate the preparation of nanogels under mild conditions. (ii) Triggering drug release by applying external stimuli such as temperature, light, ultrasound, or magnetic fields. (iii) Inducing drug release or the reverse shielding of the nanogel, thereby improving nanogel drug uptake by tumor cells in the slightly acidic tumor microenvironment; and/or (iv) increasing intracellular drug release in tumor cells in the presence of endo/lysosomal pH and/or cytosolic reducing conditions. Cheng et al. reviewed various dual- and multi-stimuli-responsive polymer nanoparticles and focused on the design and fabrication of dual- and multi-stimulus-responsive polymer NPs and their novel applications in cancer therapy for controlled drug delivery (Figure 10) [98].

#### 5.2.1. pH- and Temperature-Responsive Nanogels

Temperature and pH play important roles in the performance of nanogels. To improve the efficiency of current drug delivery systems and reduce the side effects of anticancer drugs, Aminoleslami et al. used triethylene glycol dimethacrylate (TEGDMA) as a cross-linking agent to develop dual pH- and temperature-responsive cross-linked P(VCL-co-AA) polymer nanogels by copolymerizing N-vinyl caprolactam (VCL) with acrylic acid (AA) monomers. P(VCL-co-AA) was loaded with doxorubicin as a chemotherapeutic agent with (83%) Dox loading. P(VCLco-AA) exhibited pH- and temperature-dependent behavior, and under physiological conditions, drug release in the simulated tumor area was significantly larger. The MTT assay on the HFF-2 cell line confirmed the biocompatibility and nontoxicity of P(VCL-co-AA). Furthermore, the Dox-loaded P(VCL-co-AA) nanogels exhibited higher cytotoxic effects on the Michigan Cancer Foundation 7 (MCF-7) cell line compared to the free drug, and the Dox-loaded P(VCL-co-AA) showed high efficiency for the treatment of breast cancer [99]. In one example, hybrid nanogels (HNGs) based on mesoporous silica NPs (MSNs), oligo(ethylene glycol) methacrylate, and acrylic acid (AA) or itaconic acid (IA) comonomers were synthesized using ultrasound-assisted radical precipitation. The developed HNG exhibits pH- and thermo-responsive behavior. Camptothecin (CPT) was encapsulated in HNG and cell viability of fibroblast cells (NIH 3T3) and human prostate cancer cell lines (LNCaP) was tested using standard colorimetric assays. The cytotoxicity results showed that the cell viability was more than 80% even at the highest HNG concentration. Therefore, this drug delivery system can provide the efficient controlled release of CPT in cancer cells [100].

Qian et al. developed paclitaxel (PTX)-loaded chitosan/poly(N-isopropylacrylamide) nanoparticles (NPs) for the active management and treatment of human breast cancer with KDR/Flk-1 overexpression. Chitosan (CS) is pH-sensitive, and by grafting the thermosensitive polymer poly(N-isopropylacrylamide) (PNIPAM), they were able to fabricate dual temperature- and pH-responsive CS-based NPs. This copolymer was then conjugated with an anti-breast-cancer-targeting peptide (K237), and MTT assays revealed that the K237-conjugated NPs inhibited breast cancer cell proliferation more effectively than the unconjugated NPs. They found that the peptide-functionalized NPs exhibited favorable pH- and temperature-sensitive properties, with PTX being released faster at the slightly acidic pH of the tumor microenvironment. Paclitaxel release was also enhanced by increasing the temperature. The in vitro tests revealed that the chitosan-based NPs were biocompatible. Additionally, the K237-conjugated NPs were much more toxic to cancer cells than non-cancerous cells and were taken up by tumor cells to a greater extent than the peptide-free analogs. Studies using confocal microscopy confirmed that the NPs could precisely target MDA-MB-231 human breast cancer cells overexpressing KDR/Flk-1 protein. Therefore, these results suggest that the K237-CS(PTX)-g-PNIPAM-NP system could potentially be applied for the targeted delivery and controlled release of anticancer drugs [101].

Dinari et al. synthesized a new curcumin-loaded lignin-based lignin-g-P (NIPAM- co-DMAEMA) (LNDNG) nanogel. They prepared four LNDNG systems with different lower critical solution temperatures ranging from 32 to 34, 37, and 42 °C by controlling the initial co-monomer composition and ATRP conditions. The average curcumin-loading capacity and encapsulation efficiency of LNDNG were found to be 49.69% and 92.62%, respectively. LNDNG systems have excellent properties in terms of the response to temperature and pH stimulation effects. The LNDNG system has a high tendency to absorb curcumin, and the presence of lignin may be responsible for its sustained release. Furthermore, the cumulative release amount of the loaded CUR was 65.36% after 72 h, and the cytotoxicity of the LNDNG system was observed to be at a minimal level. Therefore, these lignin-based NGs are considered to be promising, safe, and suitable vehicles for use in drug delivery [102]. Abedi et al. synthesized a nontoxic pH/thermo-responsive nanogel based on P(NIPAAm-co-DMAEMA). These nanogels were used to simultaneously efficiently and controllably deliver the anticancer drug doxorubicin (DOX) and the chemosensitizer curcumin (CUR). The prepared nanogels were used as dual pH- and thermo-responsive supports exhibiting an LCST of approximately 40 °C. The in vitro release studies showed that the high temperature and acidic pH of the cancer cells facilitated the release of the drugs from the nanocarriers. According to the findings of the cytotoxicity investigations, CUR and DOX together could more potently induce apoptosis in HT-29 colon cancer cells and exhibit higher antitumor effects than a single agent formulation or free drug. Therefore, the obtained smart nanogel could function as a nanomedicine suitable for the simultaneous administration of two drugs and achieve effective therapeutic activity in combined cancer treatment [103].

Kim et al. developed temperature- and pH-responsive β-LP (β-lapachone)-loaded PNIPAM-co-Aac nanogels. The nanogels were modified with different acrylic acid (AAc) contents, and the transition temperature of the PNIPAM nanogels increased with the AAc content. Interelectronic repulsion between the carboxyl groups of the AAc content leads to the contraction of the PNIPAM nanogel and drug release. The AAc content was found to be responsible for the controlled drug release behavior of the β-lapachone-loaded PNIPAM-co-AAc nanogels, which was effectively induced in response to pH and temperature. Moreover, at basic pH, drug release is achieved with significant synergy. The cell viability studies using fibroblasts showed that PNIPAMco-AAc20 had the best properties, showing relatively low drug release in the acidic to neutral pH range at body temperature, but increased drug release at basic pH. Therefore, this temperature- and pH-responsive PNIPAM-based nanogel may be a promising nanocarrier for intestinal-specific drug delivery potential [104]. Miceli et al. synthesized a poly (Nisopropylacrylamide) (pNIPAM)-based semi-interpenetrating polymer network (SIPN) nanogel (NG) that releases a therapeutic protein corona upon cooling. Here, poly(4-acryloylamine-4-(carboxyethyl)heptanodioic acid) (pABC) was used to semipermeabilize nanogels based on dendritic polyglycerol (dPG) and thermoresponsive poly(acryloylamine) pNIPAM. The pABC dendritic secondary network confers pH-responsive behavior to the nanogels. These SIPN-NGs are stable under physiological conditions and exhibit electrophoretic mobility tailored to body temperature. The SIPN-NG was successfully loaded with a cytochrome c (Cyt c) corona in the decayed state at (37 °C). These dual-reactive SIPN-NGs delivered Cyt c more efficiently to the cancer cells when the sample was cooled to room temperature (30 °C) compared to when the sample was kept constant at 37 °C. They achieved complete control over the reactivity of dendritic SIPN-NG as a carrier of the apoptotic reagent Cytc. Therefore, the dendritic polymer (pABC) used as a thermal trigger helped to reduce the binding affinity to Cytc and generate on-demand reactivity. Therefore, the combined use of dendritic thermoresponsive NGs as polymeric drugs and therapeutic proteins may be beneficial to reduce side effects and improve the therapeutic specificity of conventional therapeutic agents [105]. Jiang et al. developed pH/temperature-sensitive magnetic nanogels (Cy5.5-Lf-MPNA) conjugated with Cy5.5-labeled lactoferrin as a multifunctional MRI/fluorescence contrast agent for the diagnosis of glioma (the most common primary brain tumor). Due to pH/temperature sensitivity, Cy5.5-Lf-MPNA nanogels may exhibit changes in their size and hydrophilic/hydrophobic characteristics at varying pH and temperatures. Cy5.5-Lf-MPNA nanogels prolong the circulation time in the blood under physiological conditions (pH 7.4, 37 °C), which, combined with the active targeting ability of lactoferrin, has the potential to accumulate particularly in glioma tissues. Furthermore, in the acidic environment of tumor tissues (pH 6.8, 37 °C), the nanogels become hydrophobic and contract, allowing them to more easily accumulate and be taken up by tumor cells. Cy5.5-Lf-MPNA nanogels can be used to achieve MR/fluorescence imaging with high sensitivity and specificity, according to in vivo studies conducted on rats with in situ gliomas. Cy5.5-Lf-MPNNA nanogels are anticipated to be developed as sensitive and specific multifunctional MRI/fluorescence imaging agents for glioma diagnosis due to their exceptional biocompatibility [106]. The remarkable efficacy of polysaccharide-based nanogels as drug carriers for in vivo release has garnered significant attention. Polysaccharides are natural polymers derived from plants, microorganisms, algae, and some animals. They are made up of aldoses or ketoses that are linked together by glycosidic bonds. Meng et al. recently reviewed the properties and applications of nanoscale polysaccharide-based delivery carriers (including starch, pectin, chitosan, xanthan gum, and alginate), including their ability to encapsulate, protect, and deliver bioactive ingredients [107]. Li et al. recently prepared a pH and temperature dual-responsive nanogel CS/P(MAAco-NIPAM) using a radical polymerization method and used it in the loading and releasing of DOX. The TGA analysis revealed that the nanogel had high thermal stability, and SEM revealed that the dry gel particle size was around 100 nm. Upon the analysis of the drug loading and releasing mechanism of the nanogel, it was discovered that 84% of the drug was loaded into the nanogel network through electrostatic interactions. Molecular diffusion was the driving force behind the drug’s release from the gel, and the nanogel’s release efficiency reached its peak at pH = 2.0 and T = 20 °C [108].

Wen et al. created dual pH- and temperature-responsive bionanogels (DuR-BNGs) using an aqueous cross-linking polymerization method based on temperature-induced self-association. In vitro, these DuR-BNGs nanogels demonstrated excellent colloidal stability over a wide pH range. They showed enhanced release when their morphologies (volumes or ionic interactions) changed in response to individual stimuli such as high temperature and acid pH; additionally, the DuR-BNGs promoted the synergic release of encapsulated anticancer drugs (Dox) in the presence of both stimuli. The CLSM, flow cytometry, and MTT viability assay cell culture results suggest the intracellular release of anticancer drugs from the DOX-loaded DuR-BNGs into HeLa cancer cells [109].

#### 5.2.2. pH- and Glutathion (GSH)-Responsive Nanogels

Yu et al. developed a dextran-based (Dex-SS) nanogel that responds to dual pH/reduction stimuli via disulfide-containing Schiff base formation in an inverted water-in-oil microemulsion of polyaldehyded dextran and cystamine. The antitumor drug doxorubicin (DOX) was covalently attached to dextran nanogels via Schiff base linkage, and the DOX loading capacity of the Dex-SS nanogels was calculated to be 3.2%, which corresponds to a loading efficiency of 35.5%. The nanogel (DOX@Dex-SS) exhibited dual pH- and reduction (GSH)-responsive behavior in terms of the drug release. Only 9% of the DOX was released after 158 h at neutral pH (7.4) or low GSH concentration (10 μM), whereas 23.86% and 40% when the DOX@Dex-SS nanogels were incubated at pH 6.5 and 5.0 was observed to be released. It was also observed that approximately 56% and 75% of the DOX was released after 158 h of incubation with 5mM and 10mM GSH. The fastest DOX release was observed when the two stimuli of pH 5.0 and GSH 10 mM were applied simultaneously. Therefore, the DOX@Dex-SS nanogels exhibited synergistic effects when induced under dual stimulation. The results of the drug release studies indicate that DOX encapsulated in nanogels is taken up by endocytosis and induces drug release within endocytic vesicles, which is advantageous for controlling drug delivery and intracellular release. A CCK-8 assay confirmed that the DOX@Dex-SS nanogels were not cytotoxic to tumor cell lines including H1299 cells and Hela cells within the measured concentration, even at 320 μg/mL. Therefore, the developed dual pH- and GSH-responsive nanogels could be a promising microenvironment-responsive drug delivery system for cancer treatment [110].

Recently, using modified carboxymethyl chitosan (CMCS) and N-N′-bis(acryloyl)cysteamine (BAC) as cross-linking agents for the effective targeted delivery of antitumor drug DOX, pH- and glutathione (GSH)-responsive nanogel was prepared by Yang et al. The drug loading capacity (LC) and encapsulation efficiency (EE) of DOX were approximately 15.6% and 94.77%, respectively. The prepared DOX-loaded nanogel (GCCMCS-FA-DOX) has high GSH- and pH-responsive performance, and DOX release from GCMCS-FA significantly inhibits cell proliferation within the tumor microenvironment (high GSH and low pH) [111]. Mackiewicz et al. synthesized biocompatible and degradable PEG-based nanogels cross-linked with N,N′-bis(methacryloyl)cystine–(mBISS) as the redox- and pH-sensitive linker. The presence of carboxyl groups in mBISS increased the pH sensitivity of the nanogels and enabled them to bind the anticancer drug doxorubicin (DOX) through electrostatic interactions. The DOX-loaded nanogels appeared to maintain stability in physiological environments, with a drug loading capacity of approximately 8%. The release efficiency of DOX from the nanogels was influenced by the amount of the reducing agent glutathione (GSH) and the pH of the cellular environment. The addition of glutathione (GSH) degraded the nanogel particles and increased the cumulative release amount. It was observed that at pH 7.4, the lowest amount of DOX was released from the nanogels, whereas the highest cumulative release of DOX was observed under conditions similar to those found in most cancer cells (pH 5, 40 mM GSH) [112]. Despite having very strong antitumor effects, camptothecin (CPT) has limited clinical application due to its poor water solubility and severe toxic side effects. Qu et al. created a smart redox/pH dual stimuli-responsive P (CPT-MAA) prodrug nanogel that delivers CPT while also minimizing its side effects. The distillation precipitation polymerization technique was used to create P(CPT-MAA) prodrug nanogels from methacrylic acid (MAA), CPT, and N,N′-methylenebisacrylamide (Bis). CPT was attached to the nanogel using a reduction-sensitive disulfide bond in this method. The release of CPT from the prodrug nanogels was dual redox/pH-responsive due to the redox-sensitive properties of the disulfide bonds and the pH-sensitive properties of MAA. CPT release is accelerated by decreasing pH and increasing GSH concentration, promoting drug release within cells and in the tumor tissue microenvironment. Therefore, these types of prodrug nanogels may be advantageous to facilitate the invention of lots of new prodrug nanogels for clinical applications [113].

Cheng et al. synthesized pH- and oxidation-responsive diselenide-cross-linked polyurethane nanogels. First, tertiary amino groups and Br groups were added to PEGylated polyurethane (MPEG-PUBM-MPEG) micelles. Subsequently, a cross-linked nanogel with a core–shell structure was prepared using sodium diselenide (Na_2_Se_2_) as a cross-linking agent. The dynamic light scattering (DLS) analysis and atomic force microscopy (AFM) measurements revealed that the diselenide-cross-linked nanogels have improved colloidal stability compared to the non-cross-linked micelles. The drug loading amount and drug loading efficiency of the nanogels were determined to be 18.5% and 76.3%, respectively, using indomethacin (IND), a hydrophobic chemotherapeutic agent. The nanogels exhibited pH-induced swelling process and oxidation-induced degradation properties, and drug release from the nanogels was enhanced under acidic and oxidative conditions. The cumulative release of IND at pH 5.0 with 50 mM H_2_O_2_ was almost 80% in the first 20 h, whereas at pH 5.0 with 100 mM H_2_O_2_, almost all the drug was released from the nanogel in 30 h. This demonstrates the synergistic effect of pH- and oxidation-induced double dissociation in diselenide-cross-linked nanogels [114]. Zhang et al. prepared pH/redox-reactive xanthan gum nanogels from xanthan gum cross-linked with cystaminetetraacylhydrazine (CTA) in a one-step process. As a model drug, doxorubicin was loaded into the nanogels at a rate of up to 17.88%. An increase in the cumulative release was observed as the pH changed from 5.0 to 7.4. Drug release was also accelerated in the presence of the reducing agent glutathione (GSH), with steady-state cumulative releases up to 72.1, 55.2, and 38.3% at pH 5.0, 6.5, and 7.4 observed in 10 mM GSH solutions. In the absence of GSH, the steady-state cumulative release was only 44.9, 37.0, and 29.9% at pH 5.0, 6.5, and 7.4, respectively. Therefore, the nanogels exhibited good dual pH/redox responsiveness. MTT studies have shown that nanogels are biocompatible and non-cytotoxic and could potentially be usedas carriers for the targeted delivery of anticancer drugs [115].

#### 5.2.3. Temperature- and Glutathion (GSH)-Responsive Nanogels

Recently, Rao et al. developed an intracellularly induced temperature-sensitive and redox-sensitive doxorubicin (Dox)-encapsulated Cys-BIS-P (VCL-HEA) nanogel for anticancer drug delivery. To obtain the Cys-BIS-P(VCL-HEA) nanogels, a three-dimensional polymer network was made of poly(vinylcaprolactam) (PVCL) with disulfide bonds. Cys-BIS-P(VCL-HEA) has a high Dox drug loading of 49% and is stable under extracellular conditions (pH 7.4 at 37 °C). In the presence of typical intracellular glutathione concentrations, the model drug Dox is released from the nanogel through disulfide bond rupture. The in vitro results showed that the nanogels promoted internalization into HepG2 cancer cells by breaking disulfide bonds within the nanogel network and releasing Dox near the cell nucleus, destabilizing the intracellular cytosol, and it has been confirmed that it can effectively kill cancer cells [116]. In one example, degradable core–shell nanogels of different compositions were synthesized from PNIPAM (N-isopropyl methacrylamide) and PNIPMAM (N-isopropyl methacrylamide). The agglomeration behavior of these particles’ aqueous dispersions in the presence of salts can be tuned by varying the monomer ratio. The inclusion of the degradable cross-linker bis(acryloyl)cystamine (BAC) allows the nanogels to degrade in the presence of reducing agents. At pH 10 and a reducing agent concentration of 150 mM, the PNIPAM particles were only 73% degradable, whereas the PNIPMAM-containing particles were almost completely degradable (97.5%). These degradation levels were also observed at physiologically relevant pH values and reducing agent concentrations of pH 7 and 10 mM. The advanced degradation and ability to tune the thermal response behavior of the nanogels demonstrated in this study will be useful for the application of nanogels in biological applications [117].

Li et al. developed a nanogel (I/D@NG) formulated with the infrared dye indocyanine green (ICG) and the anticancer drug doxorubicin (DOX). I/D@NG demonstrated an improved photothermal effect in vitro, and the thermal effect induced by NIR light notably improved the in vivo process of I/D@NG. NIR irradiation at the tumor site was found to significantly increase the circulation time in the blood, tumor accumulation, tumor penetration, and uptake of I/D@NG by cells. Furthermore, after being taken up into the cancer cells, I/D@NG escapes from lysosomes through lysosomal disruption by singlet oxygen, and then DOX enters the nucleus in response to the intracellular photothermal effects caused by high GSH and NIR light. This nanoplatform overcomes all the physiological barriers of therapeutic agents and efficiently exerts the synergistic effect of hyperthermic chemotherapy, thereby achieving significant anticancer effects in vivo [118].

#### 5.2.4. Other Dual Responsive Nanogels

Diabetes has emerged as one of the most serious threats to human health, and numerous insulin delivery systems have been developed to treat diabetes in recent decades. To improve glucose-responsive insulin delivery, Li et al. developed dual-sensitive (glucose and H_2_O_2_) nanogels to enhance glucose-responsive insulin delivery. Polymer nanogels were synthesized using poly (ethylene glycol) and poly (cyclic phenylboronic ester) of dual sensitive glucose and H_2_O_2_ groups. Glucose oxidase (GOx) was incorporated into the nanogel, which generated H_2_O_2_ at high glucose levels and improved glucose response and insulin release. For the GOx-free nanogels in the presence of 0, 1, and 4 g L^-1^ glucose, the cumulative release rates of insulin were observed to be 16%, 38%, and 59% at 30 h, respectively. Furthermore, the release rates of 0, 0.1, and 0.5 mM H_2_O_2_ at 10 h were 13.2%, 21%, and 29%. The nanogels loaded with 1 wt% GOx/insulin showed the accelerated release of insulin with a cumulative release of 57% and 78% at 30 h in the presence of 1 and 4 g L-1glucose, respectively. Therefore, the introduction of GOx effectively enhanced the glucose responsiveness and promoted insulin release. The nanogels demonstrated long circulation in the blood, rapid glucose response, and good biocompatibility. Subcutaneous insulin administration to diabetic mice using insulin/GOx-loaded nanogels resulted in an effective hypoglycemic effect when compared to the insulin-only nanogels. Therefore, these types of nanogels will be promising candidates for future diabetes treatment [119].

Wang et al. created dual-responsive PEG-chitosan@CD hybrid nanogels for controlled drug release and synergistic therapy by polymerizing nonlinear polyethylene glycol (PEG), chitosan, and graphite carbon dots (CDs). CDs embedded in PEG-chitosan@CD hybrid nanogels increase the hybrid nanogels’ loading capacity for hydrophobic anticancer drugs while also serving as excellent confocal and TPF cell imaging probes and fluorescent pH-sensing probes. Chitosan can cause pH-dependent swelling and deswelling of hybrid nanogels, allowing for pH-controlled drug release over a physiologically relevant range. The hybrid nanogels can penetrate into the intracellular region, and the pH-sensitive volume phase transition of the hybrid nanogels enables in situ pH-controlled drug release. Furthermore, under NIR light irradiation, the thermosensitive nonlinear PEG network can trigger drug release via local heat generated by the embedded CDs. Because of their strong synergistic effects, the hybrid nanogels demonstrated high therapeutic efficacy in vitro. The nanogel is nontoxic and can effectively deliver drugs to the kidney and liver tissues of mice. Therefore, these multifunctional nanogels have a lot of promise for in vivo medical diagnosis and treatment [120]. Through a concise intracellular drug delivery method, Chen and co-workers developed a dual sensitive supramolecular nanogel. To investigate pH- and reduction-induced drug release, pH-insensitive benzoic acid (BA) and benzimidazole (BM) were grafted onto dextran and thiol-b-cyclodextrin (b-CD-SH). At the tumor site (low pH (approximately 6.8) or high GSH concentration), the cross-linking disulfide bonds are cleaved, the inner core of the nanogel is released, and the nanogel swells to release the drug. Doxorubicin (DOX) was used as a model drug to test the feasibility of using these nanogels for intracellular drug delivery in cancer chemotherapy. These DOX-loaded nanogels were found to have higher DOX release rates under lower pH or higher GSH concentrations [121]. Lei et al. prepared a redox/temperature-reactive zwitterionic nanogel P(VCL-ss-DMAPS) by copolymerizing N-vinylcaprolactam (VCL) and 2-(methacryloyloxy) ethyldimethyl-(3-sulfopropyl) ammonium hydroxide (DMAPS) by aqueous precipitation polymerization. Zwitterionic nanogels play an intelligent role in the application of stealth nanocarriers with extremely high colloidal stability and excellent antifouling properties. In addition, they have excellent redox degradability and adjustable temperature sensitivity. Under physiological conditions, nanogels containing the anticancer drug doxorubicin (DOX) exhibited low DOX leakage (only 23.8% in 24 h), while the highest DOX release (93.4% in 24 h) was observed in PBS 7.4 with 10 mmol GSH at a temperature just higher than the VPTT of P(VCL-ss-DMAPS-20) nanogel (42 °C) [122]. Because of its safety, non-invasiveness, and ease of control, ultrasound is used in the stimulus-responsive drug delivery system “DDS”. A stimuli-responsive dual redox/ultrasound nanogel system was developed to precisely control drug release. To begin, the thermoresponsive PEIm-PNIPAMn-PEIm copolymer was prepared and self-assembled into micelles in aqueous solution at temperatures above its LCST. The PEI gel shell was then cross-linked with BACy containing disulfide to form spherical nanogels. The nanogels were infused with ultrasonic-responsive perfluorohexane (PFH), yielding a dual ultrasound/redox-responsive drug delivery system. Although these nanogels were found to have good structural stability, the PEI gel shell disintegrated after 1 h of exposure to GSH. The drug release was temperature- and PNIPAM-size-dependent, and it was discovered that the drug release reached 90% within 10 min under the synergistic effect of redox/ultrasound. Furthermore, the nanogel demonstrated good biocompatibility with HEK293 and Huh7 cells. The DOX-containing nanogels were more toxic to the tumor cells than to normal cells. These findings suggest that the fabricated dual stimuli-responsive nanogels have promising application prospects for controlled drug delivery/release [123].

Recently, dual ROS/electroresponsive nanogels based on phenytoin (PHT) prodrugs were developed to target epileptic foci to reconstitute defective circuits and inflammatory microenvironments. Zhou et al. combined the model drug PHT with an electroresponsive group (sodium sulfonate, SS) and an epileptic focus detection group (α-methyl-tryptophan, AMT) to form a nanogel. The nanogel was observed to target epileptic foci and release PHT in response to electrical stimulation with high concentrations of ROS in the microenvironment, inhibiting hyperexcited circuits. Meanwhile, nanogels can also reduce oxidative stress and reduce inflammation in the microenvironment by eliminating ROS. Nanogels produced in this way enablethe more targeted accumulation and on-demand release of active ingredients for antiepileptic treatment [124]. A summary of the dual stimuli-responsive nanogels is shown in Table 2.

### 5.3. Multi-Stimuli-Responsive Nanogel Systems

In addition to single- and dual-responsive nanogels, several multi-responsive nanogels have been recently developed. Multi-stimuli-responsive nanogels allow for extremely effective targeting and therapeutic capabilities, such as chemotherapy and photodynamic therapy. Recently, Pillarisetti et al. fabricated multi-stimuli-responsive alginate nanogels (abbreviated as MSAN) by conjugating oxidized alginate (OA) with 4-mercaptophenylboronic acid and pheophorbide A (a hydrophobic photosensitizer) and conjugating with adipic acid dihydrazide. Doxorubicin, an anticancer drug, was encapsulated in the nanogels, and the drug loading was approximately 10% and the encapsulation efficiency was 43%. The release behavior of pheophorbide A and doxorubicin was sensitive to pH and redox. Disulfide bonds stimulate MSAN redox, whereas pH changes cause hydrazone bond cleavage in hydrazide-functionalized pheophorbide-A(PA). MSAN’s cancer-cell-specific toxicity was studied in mouse breast cancer 4-T1 and melanoma B16F10 cell lines. MSAN can deliver PA and DOX to cancer cells at the same time, while decreasing the redox potential, which enhances PDT by increasing the generation of reactive oxygen species (ROS). These results suggest that pH stimulation not only decreases the redox potential and increases the photodynamic effect, but also promotes the release of DOX. Therefore, these multi-stimulus-responsive alginate nanogels (MSANs) may increase toxicity in breast cancer and melanoma [125].

Li et al. synthesized thermoresponsive phospholipid–poly(N-isopropylacrylamide) (PL–PNIPAM) conjugates. To generate cross-linkable functional groups, NIPAM was copolymerized with N-succinimidyl acrylate (NSA). At 37 °C (above the lower critical solution temperature), size-controllable nanogels were created by cross-linking the PL–P(NIPAM-co-NSA)/Ca^2+^ complex with cystamine. These synthesized nanogels exhibit multi-responsive behavior (temperature/pH/reduction). Doxorubicin (EE=66.7%, LC=6.2%) and proteins (EE=65%, LC=6.1%) can be efficiently loaded into the nanogels. Both low-isoelectric-point proteins (BSA and GOx) and high-isoelectric-point proteins (RNase A, CC, and Lys) were efficiently immobilized on the nanogels by adjusting the pH of the medium. Immobilized Rnase A, BSA, and Gox retained the biological activity of the protein. These multi-stimuli-responsive nanogels can provide a versatile platform for drug/protein delivery. These nanogels offer great potential in biotechnology and drug/protein delivery due to their triple responsive behavior [126]. For example, Huang et al. fabricated multi-stimulus-responsive (pH/redox/UV irradiation) nanogels based on self-assembled polymer micelles with a six-armed star-shaped copolymer of 6AS-PCL-PAAPPEGMA. Nanogels with spherical-structured 6AS-PCL-PAAPPEGMA with dimensions larger than about 150–200 nm were obtained by complexing iron ions (Fe^3+^) and carboxyl groups in polymer micelles. The nanogels were then encapsulated in the anticancer drug doxorubicin (DOX) with a drug loading content (DLC) of 12.04% and demonstrated promise as an anticancer drug carrier for “on-demand” drug delivery. It was observed that only 17.6% of the drug was released after 110 h at pH 7.4, while DOX release was 43.3% at pH 5.0. Additionally, the cumulative DOX release was increased up to 52.2% when 10 mM GSH redox stimulation was introduced for 110 h. The fastest and highest DOX release was observed (up to 82.1% after 116h) when UV irradiation was applied at simulated lysosomal pH and redox conditions, as Fe^3+^ was reduced to Fe^2+^ and the nanogels containing DOX decomposed. The DOX-loaded 6AS-PCL-PAA-PPEGMA nanogels exhibited good degradability, biocompatibility, and multi-stimuli responsiveness to pH/redox/UV radiation. Furthermore, the DOX-containing nanogels could not only be rapidly absorbed by HepG2 cells but also inhibit cell proliferation. Therefore, the 6AS-PCL-PAA-PPEGMA nanogel is a promising drug carrier for cancer treatment applications [127].

Chen et al. formed a photo-, pH-, and redox-sensitive poly (acrylic acid methacrylate-co-spiropyran) nanogel cross-linked with disulfide-containing N,N-bis(acryloyl)cystamine. When exposed to UV light or low pH, the hydrophobic spiropyran in the nanogel isomerizes into hydrophilic merocyanine, causing the nanogel to swell. Through the addition of the reducing agent dithiothreitol (DTT), the nanogels were disrupted by the oxidative cleavage of the BAC disulfide cross-links. The anticancer drug Dox can be loaded into the nanogel with a loading capacity of 18%, which will be released from the nanogel in response to UV light, pH, and DTT stimulation. They reported that the SP-based nanogel containing the anticancer drug Dox could effectively kill cancer cells, and the effect was enhanced when the nanogel was irradiated with UV light. Therefore, thistype of multi-responsive nanogel offers great potential for controlled drug delivery and cellular fluorescence imaging [128]. Cao et al. synthesized multi-stimuli-responsive nanogels from hydrophilic, thermosensitive, pH-sensitive poly(2-(dimethylamino) ethyl methacrylate) (PDMAEMA) and photocleavable o-nitrobenzyl linkage (ONB). The selective release of a variety of target cargoes with different solubility properties (hydrophobic and hydrophilic) was demonstrated by the nanogels. In this study, hydrophobic cargoes were introduced into the hydrophobic interior of the nanogel in a noncovalent manner (the loading content of hydrophobic cargoes was 17.3%) while hydrophilic cargoes (the loading content of RhB in the nanogel is 0.1%) covalently bonded through a redox-degradable disulfide junction with the polymer nanogel PDMAEMA. The cumulative release of the hydrophobic cargo increased from 41% to 86% upon irradiation at power densities of 3 mW/cm^2^ and 12 mW/cm^2^ within 23 min, respectively. Over a 16 h period, only 8% of the hydrophobic cargo molecules were released at pH 7.4, while 59% were released at pH 4.0. The nanogel solution was exposed to UV light (12 mW/cm^2^) for only 11 min after 23 h of incubation, and a burst release of 96% of the hydrophobic cargo molecules was observed, achieving pH-coupled stimulation and UV rays. The controlled release of the hydrophilic cargo RhB from multi-stimuli-responsive nanogels was achieved at various reducing agent concentrations (DL-dithiothreitol), with the amount of the released hydrophilic cargo molecules increasing from 5% to 18.5% as the DTT concentration increased from 5 mM to 20 mM. The effects of the reducing agent DTT on the release of hydrophobic cargoes, as well as the effects of temperature, UV radiation, and pH, were also investigated, and it was discovered that the redox reagent has little effect on the release of the hydrophobic cargoes. Similarly, at high temperatures, UV irradiation, and low pH, a small fraction of hydrophilic cargo may be released. As a result, under different stimulation conditions, different cargoes with different hydrophilic/hydrophobic properties can be selectively released [129].

Chen et al. fabricated self-healing nanocomposite hydrogels via a benzoxaborole-diol complex on the nanointerface. Temperature-responsive nanogels with galactose residues on the nanosurface were successfully synthesized and used as fillers and macroscopic cross-linkers in hydrogels prepared with poly (DMA-st-MAABO) linear hydrophilic copolymer containing benzoxaborole fragments. Benzoxaborole’s low pKa value (7.2) allows for rapid network formation at physiological pH (7.4), which is critical for biomedical applications. The resulting hydrogel has excellent self-healing properties and a wide range of reactivity to pH, sugar, ROS, ATP, H_2_O_2_, and temperature [130]. Manzanares et al. reported the use of a curcumin (CUR) nano formulation with cationic nanogels for the treatment of colon cancer. They used large-scale polymerization to create stimuli-responsive cationic nanogels based on the surfactant-free emulsion polymerization of N,N′-diethylaminoethyl methacrylate (DEAEM) and poly(ethylene glycol) methacrylate (PEGMA). During the preparation of these nanogels, different cross-linking agents are used. EGDMA (ethylene glycol dimethacrylate), DVA (divinylacetal), BAC (N,N′-bis (acryloyl) Cystamine), and FDAC (fluorescein diacrylate) were used, which had a significant impact on their properties. EGDMA provides stability to the nanogels and the nanogels are very stable at room temperature for up to 18 months, DVA provides the acid degradation reaction, BAC provides both acid degradation behavior, and GSH and FDAC provide the fluorescent nanogels, i.e., nanogels that can be monitored inside cells by fluorescence microscopy. The nanogels were confirmed to have a uniform size distribution from 51 to 162 nm with PDI 0.138, a spherical shape, and a core–shell morphology using dynamic light scattering and electron microscopy. These nanogels contain curcumin (CUR) and, depending on the cross-linker used in their synthesis, are sensitive to pH, temperature, and a redox environment. The nanogels loaded with CUR were found to be stable for up to 30 days under physiological conditions, and the nanogels cross-linked with BAC degraded in 60 min in buffer (pH 7.4) containing 3 mM glutathione, while the nanogels cross-linked with DVA cross-linker degraded in 10 min (pH 6). In vitro studies on a human colon cancer cell line (HCT-116) revealed that CUR and degradable nanogels work synergistically. Thus, nanogels containing curcumin and cross-linked with DVA or BAC show great potential in colon cancer treatment [131].

Pan et al. synthesized multi-redox-, temperature-, and pH-responsive nanogels based on tailor-modified sugarcane bagasse cellulose (SBC). These nanogels were formed by the in situ radical copolymerization of monocarboxylic methacrylate sugarcane bagasse cellulose (MAMC-SBC) and N-isopropylacrylamide (NIPAM) in the presence of cysaminebisacrylamide (CBA) as a disulfide cross-linker. MAMC-SBC formed the main structure of the nanogel and NIPAM and CBA provided the temperature and redox reactivity to the nanogel, respectively. Doxorubicin hydrochloride (DOX), as a model drug, was effectively loaded into the nanogels with a drug loading efficiency of up to 82.7%. Furthermore, drug release can be triggered by reducing agents, pH, and temperature, which is due to the multi-responsive activity and synergistic effects of nanogels. Therefore, the prepared multi-responsive nanogels showed a good drug loading efficiency and showed great potential for controlled drug release [132]. A summary of the multi stimuli-responsive nanogels is shown in Table 3.

## 6. Concluding Remarks

Herein, we examined, discussed, and reviewed the progress of stimulus-responsive nanogels. Different types of single- and multi-reactive nanogels are being investigated for controlled drug delivery as the field of drug delivery evolves. The stability, hydrophilicity, and biocompatibility of stimuli-responsive nanogels make them significantly superior to other NPs in drug delivery; however, they have not yet reached their full potential in biomedical applications. The current challenges in drug delivery involve the development of mechanisms capable of delivering sufficient quantities of drugs efficiently and effectively overcoming various physiological barriers to reach the treatment site. The most significant impact of nanotechnology on drug delivery is the development of less toxic cancer treatments that reduce side effects, increase dosage and cellular absorption, and ensure sustained release. There is still remarkable work to be done, including the development of advanced stimulus-responsive and drug-loaded nanogels. Furthermore, chemotherapy based on stimuli-responsive nanogels can be combined with other treatments, such as photodynamic therapy, photothermal therapy, and radiotherapy, thereby providing an innovation for cancer treatment. Only with continued development and innovation can the great potential of using stimuli-responsive nanogels inbiomedical applications be sustained.

## Figures and Tables

**Table 1:** Summary of the individual stimuli-responsive nanogels for biomedical applications.

Stimuli	Nanogels	Drug/Cargo	Therapeutic Field	Ref.
pH	Methacrylic acid and bovine serum albumin (PMAA-BSA)-based nanogel	chloroquine (CQ)	malaria and cancer	[49]
	polyvinylpyrrolidone (PVP)-based nanogel	5-fluorouracil (5-FU)	colorectal cancer	[50]
	chitin nanogels	doxorubicin (DOX)	cancer therapeutic agent	[51]
	dextrin nanogels cross-linked with formaldehyde (FDNGs) and glyoxal (GDNGs)	DOX	colorectal cancer therapy	[52]
	chitosan-based nanogel (FCNGL)	5-Fluorouracil	melanoma animal tumor	[53]
	hyaluronic acid nanogels (HA-mPEG-Diet nanogels)	Cytochrome C (CC)	protein delivery and cancer therapy	[54]
	hyaluronate nanogels	bortezomib (BTZ)	chemotherapy	[55]
	nanogels based on ortho ester-modified PEG	DOX	anti-tumor	[56]
	chitin-based nanogel Dox–chitin–PLA CNGs	DOX	liver cancer	[57]
Temperature	activated poly(N-isopropylacrylamide) (PNIPAM) nanogels and N-acryloylglycinamide (NAGA)-based nanogel	-	tissue engineering	[60]
	(N-isopropylacrylamide)-acrylic acid copolymer (NIPAM-co-AA) and iohexol-based nanogel	-	digital subtraction angiography	[61]
	poly(N-isopropylacrylamide) (PNIPAM)-based nanogel	Timolol maleate (TM)	contact lenses	[62]
	TCN-TS-NC nanocargo	topotecan (TCN)	tumor therapy	[63]
	dendritic Polyglycerol- N-isopropylacrylamide (dPG-NIPAm)-based nanogels	coumarin 6 dye	controlled delivery of drugs through the hair follicle	[64]
	poly (N-isopropylacrylamide) and poly(3,4-ethylenedioxythiophene) based nanogel (PEDOT@PNIPAAm)	curcumin (Cur)	antioxidant and antibacterial	[65]
	poly (N-vinylcaprolactam) nanoparticles (νPVCL)	sodium diclofenac	topical delivery of multiple drugs	[66]
	nanogel based on chitosan and N-isopropylacrylamide with acrylamide blended CTS–poly (NIPAAm-co-AAm5.5)	Paclitaxel (PTX)	anticancer drugs and hyperthermia	[67]
Bioreduction/redox	Se-S alginate nanogel (MDSeSAN-gel)	DOX	cancer therapy	[69]
	Gelatin/Silica-Aptamer -based nanogels (Apt-GS/siRNA)	siRNA	tumor therapy	[70]
	2-((2-hydroxyethyl)disulfanyl)ethylMethacrylate (HSEMA)-based nanogel	R848	cancer immunotherapy	[71]
	Dextran and β-cyclodextrin (βCD)-based nanogel with bisadamantine as cross-linker	DOX	breast cancer	[72]
	nanogels (DPH NGs) based on reductive cross-linking of purpurin 18 and 10-hydroxycamplothecin	hydroxycamplothecin (HCPT)	chemotherapy	[73]
Biomolecule (glucose/enzyme, etc.)	3-acrylamidophenylboronic acid (AAPBA), 2-(acrylamido) glucopyranose (AGA) and boron dipyrromethene (BODIPYMA)-based nanogel p(AAPBA-AGA-BODIPYMA)	Insulin	self-regulated insulin delivery	[76]
	N-acryloyl-3-aminophenylboronic acid, poly(ethylene glycol) diacrylate, pentaerythritol tetra (3-mercaptopropionate), and methoxyl poly(ethylene glycol) acrylate-based nanogel	Alizarin red S (ARS) and insulin	self-regulated drug delivery	[77]
	Glucose oxidase (GOX) polymer nanogel	Glucose oxidase (GOX)	synergistic cancer starving and oxidation therapy	[78]
	acetalated-dextran polymeric nanoparticles	Insulin	controlled insulin delivery for diabetes	[79]
	G4 PAMAM (polyamidoamine) dendrimers-based nanogel	DOX	cancer therapy	[80]
	photo-cross-linked nanogels (EPNGs)	cytochrome c (CC)	cancer therapy	[81]
	hyaluronated starch nanogels	Docetaxel (DTX	tumor therapy	[83]
	poly(ethylene glycol)–poly(e-caprolactone), diketopyrrolopyrrole, fluconazole-based nanogel (PGL-DPP–FLU NPs)	Fluconazole (FLU)	antifungal	[84]
	ketone-functionalized water-soluble pullulan derivative and carbonic dihydrazide-based nanogel	-	bacterial enzyme detection	[85]
Light	nanogel based on acrylamide and acrylonitrile copolymer cross-linked with nickel-bis(dithiolene) complex	-	NIR light-controlled drug delivery and photothermaltreatment	[89]
	anionic azobenzene-functionalized hyaluronic acid and cationic poly diallyl dimethylammonium chloride polymers-based nanogel	DOX	UV-controlled intracellular drug release in cancer chemotherapy	[90]
	hyaluronic acid-g-7-N,N-diethylamino-4-hydroxymethylcoumarin (HA-CM)-based nanogel	DOX	NIR and UV-responsive drug release in cancer chemotherapy	[91]
	nanogel based on chitosan–poly(N-isopropylacrylamide) (PNIPAM) and modified with gold and magnetic nanoparticles	-	visible light-sensitive drug carrier	[92]
	pentaerythritol poly(caprolactone)-bpoly (acrylic acid)-based nanogel	DOX	tumor therapy	[93]

**Table 2:** Summary of the dual stimuli-responsive nanogels for biomedical applications.

Stimuli	Nanogels	Drug/Cargo	Therapeutic Field	Ref.
pH/temperature	N-vinyl caprolactam (VCL) and acrylic acid (AA)-based nanogel cross-linked with triethylene glycol dimethacrylate (TEGDMA)	Doxorubicin (Dox)	breast cancer	[99]
	Mesoporous silica nanoparticles (MSNs), oligo(ethylene glycol) methacrylate, and acrylic acid (AA) or itaconic acid (IA)-based nanogel	Camptothecin (CPT)	cancer therapy	[100]
	chitosan/poly(N-isopropylacrylamide) nanoparticles	paclitaxel (PTX)	breast cancer	[101]
	lignin-based lignin-g-P(NIPAM-co-DMAEMA) (LNDNG) nanogel	curcumin	drugs delivery	[102]
	N-Isopropylacrylamide (NIPAAm) and N,N-dimethyl-aminoethyl methacrylate (DMAEMA)-based nanogel P(NIPAAm-co-DMAEMA)	doxorubicin and curcumin	combined cancer therapy	[103]
	Poly-N-isopropyl acrylamide and acrylic acid-based nanogels (PNIPAM-co-AAc)	β-lapachone	intestine-specific drug delivery	[104]
	dendritic polyglycerol (dPG), poly(Nisopropylacrylamide) (pNIPAM) and poly (4-acryloylamine-4-(carboxyethyl) heptanodioic acid) (pABC)-based nanogel	cytochrome c (cyt c)	release of a therapeutic protein	[105]
	magnetic nanogels conjugated with Cy5.5-labled lactoferrin (Cy5.5-Lf-MPNA nanogels)	Cy5.5	glioma (brain tumor)	[106]
	chitosan-functionalized nanogel CS/P(MAAco-NIPAM)	DOX	drug delivery	[108]
	cellulose-based nanogel (DuR-BNGs)	DOX	cancer therapy	[109]
pH/bioreduction	dextran-based (Dex-SS) nanogels	DOX	cancer therapy	[110]
	modified carboxymethyl chitosan (CMCS)-based nanogel using N-N′-bis(acryloyl)cysteamine (BAC) as cross-linking agents	DOX	tumor therapy	[111]
	PEG-based nanogel cross-linked with linker N,N′-bis(methacryloyl)cystine–(mBISS).	DOX	cancer therapy	[112]
	methacrylic acid (MAA), Camptothecin (CPT) and N,N′-methylenebisacrylamide (Bis)	Camptothecin (CPT)	tumor therapy	[113]
	Diselenide-cross-linked polyurethane nanogel	indomethacin (IND)	chemotherapy	[114]
	Xanthan gum based nanogelscross-linked by cystamine tetra-acylhydrazine (CTA)	DOX	anti-cancer	[115]
Temperature and redox	Cys-BIS-P (VCL-HEA) nanogel	DOX	anti-cancer	[116]
	PNIPAM (N-isopropyl methacrylamide) and PNIPMAM (N-isopropylmethacrylamide)-based nanogel using bis(acryloyl)cystamine (BAC) as cross-linker	-	nanomedicine	[117]
	Indocyanine green- and anticancer drug Doxorubicin-loaded nanogels (I/D@NG)	DOX	thermo-chemotherapy	[118]
Glucose/H_2_O_2_	poly (ethylene glycol) and poly (cyclic phenylboronic ester) and Glucose oxidase (GOx)-based nanogel	insulin	diabetes therapy	[119]
pH/light	poly(ethylene glycol) (PEG), chitosan, and graphitic carbon dots (CDs)-based nanogel	DOX	synergistic cancer therapy	[120]
pH/reduction	dextran-grafted benzimidazole (Dex-g-BM) and thiol-β-cyclodextrin-based nanogel	DOX	cancer chemotherapy	[121]
Temperature/redox	zwitterionic P(VCL-ss-DMAPS) nanogel based on N-vinylcaprolactam and 2-(methacryloyloxy) ethyldimethyl-(3- sulfopropyl) ammonium hydroxide	DOX	tumor therapy	[122]
Redox/ultrasound	PEIm-PNIPAMn-PEIm gel cross-linked by disulfide-containing BACy followed by perfluorohexane (PFH) incorporation	DOX	tumor therapy	[123]
ROS/electroresponsive	sodium sulfonate and α-methyl-tryptophan based nanogel	phenytoin (PHT)	antiepileptic treatment	[124]

**Table 3:** Summary of the multi-stimuli-responsive nanogels for biomedical applications.

Stimuli	Nanogels	Drug/Cargo	Therapeutic Field	Ref.
pH/redox/light	oxidized alginate (OA), 4-mercaptophenylboronic acid, pheophorbide A and adipic acid dihydrazide-based nanogel	DOX	breast cancer and melanoma	[125]
	polymer micelles self-assembled by six-arm star-shaped copolymer of 6AS-PCL-PAAPPEGMA-based nanogel	DOX	cancer therapy	[127]
	poly(acrylic acid-co-spiropyran methacrylate) cross-linked by disulfide-containing N,N-bis(acryloyl)cystaminenanogel	DOX	delivery and fluorescence cell imaging	[128]
pH/temperature/light	poly (2-(dimethylamino) ethyl methacrylate) and hydrophobic photocleavable o-nitrobenzyllinkage-based nanogel	Coumarin 102 and rhodamine B (RhB)	tissue engineering and combination chemotherapy	[129]
pH/ROS/sugar/ATP/ temperature	hydrogels based on interfacial polymer nanogel benzoxaborolate	self-healing	tissue engineering and controlled drug delivery	[130]
pH/temperature/redox	N,N′- diethylaminoethyl methacrylate and poly(ethyleneglycol) methacrylate-based nanogel cross-linked with EGDMA (ethylene glycol Dimethacrylate), DVA (divinylacetal), BAC (N,N′-bis(acryloyl) cystamine) and FDAC (fluorescein diacrylate)	curcumin	colon cancer therapy	[131]
	methacrylated monocarboxylic sugarcane bagasse cellulose (MAMC-SBC) and N-isopropylacrylamide (NIPAM) based nanogel cross-linked with cystaminebisacrylamide (CBA)	DOX	cancer therapy	[132]
